# A suite of agronomic factors can offset the effects of climate variability on rainfed maize production in Kenya

**DOI:** 10.1038/s41598-022-19286-2

**Published:** 2022-10-03

**Authors:** Kevin Ong’are Oluoch, Hugo De Groote, Zachary M. Gitonga, Zhenong Jin, Kyle Frankel Davis

**Affiliations:** 1 Department of Plant and Soil Sciences, University of Delaware, Newark, DE USA; 2 International Maize and Wheat Improvement Center (CIMMYT), Nairobi, Kenya; 3 Department of Bioproducts and Biosystems Engineering, University of Minnesota - Twin Cities, Saint Paul, MN USA; 4 Institute on the Environment, University of Minnesota - Twin Cities, Saint Paul, MN USA; 5 Department of Geography and Spatial Sciences, University of Delaware, Newark, DE USA

**Keywords:** Climate sciences, Environmental social sciences, Climate-change adaptation, Climate-change mitigation

## Abstract

Achieving food security in sub-Saharan Africa (SSA) is a multidimensional challenge. SSA reliance on food imports is expected to grow in the coming decades to meet the population's demand, projected to double to over 2 billion people by 2050. In addition, climate change is already affecting food production and supply chains across the region. Addressing these multiple food security challenges will necessitate rapid enhancements in agricultural productivity, which is influenced by a host of demographic, agronomic, and climatic factors. We use statistical approaches to examine rainfed maize in Kenya, where maize cultivation and consumption are widespread and central to livelihoods and national food security. We find that improving a suite of agronomic factors, such as applying fertilizer, planting certified seeds, and extension services, will have a greater effect on rainfed maize productivity than demographics and can offset the effects of climate change. These findings could also offer insights into similar challenges for other crops in Kenya and other SSA countries.

## Introduction

Sub-Saharan Africa (SSA) faces multiple food security challenges. Currently, 22% of the people living in SSA are undernourished^1^, and the region relies on large amounts of food imports to meet local demand^2^. In addition, climate change is already affecting food production and supply chains across SSA^3,4^. Compounding these issues, the population of SSA is projected to double from about 1 billion people currently to over 2 billion by 2050^5^, which will make it home to one in four people globally^6^. Addressing current food security challenges while reducing import reliance, meeting rising food demand, and coping with the effects of climate change will necessitate rapid enhancements in agricultural productivity across the SSA region, particularly for staple cereals, which constitute 50% of the current average calorie intake in developing countries^2,7,8^. While past studies have demonstrated that many parts of SSA do indeed possess an immense potential to increase cereal yields through improved access to irrigation, fertilizers, and other inputs^9,10^, a host of demographic, agronomic, and climatic factors converge to exercise influence on yield outcomes. Yet, these factors are rarely considered together in studies evaluating the extent to which SSA farmers can feasibly enhance cereal yields. Such integrated considerations are essential in SSA where most farmers practice rainfed agriculture on relatively small family-owned plots—80% of farms are less than 2 hectares^11^—and grow a diversity of crops that serve multiple purposes, including supporting household nutrition, diversifying their marketable goods, and mitigating drought risk^12^.

The demographics of SSA farmers differ from those of developed countries, where large-scale agriculture is more widely practiced. Previous work has examined how specific demographic characteristics can influence yield outcomes for SSA farmers^13,14^. For instance, one recent study showed that labor supply(in hours per day) per worker—who are household members—is low in SSA because farmworkers engage in other economic activities or primarily work in another sector of the economy^14^. Consequently, smallholder farms often have lower per capita income as compared to larger farms^15^, smallholder yield has been found to inversely correlated with farm size in Kenya^12,16^. Other studies examining the relationship between yield and gender of smallholder farmers have described how male farmers may achieve better yields outcomes than female farmers because cultural norms give them better land ownership and farming management rights (to acquire inputs, labor, and extension services)^13,15,17^. Other work on smallholder demographics found that educated farmers have better uptake of improved technologies^13,15^. All this previous work indicates that the demographics of smallholder farmers are essential to consider in developing effective strategies to improve food production in SSA.

Climate variability and change continue to disrupt rainfed agriculture in SSA primarily through changing rainfall patterns, rising annual temperatures, and increasing extreme events^18^. Extensive research has examined the relationship between climatic factors and rainfed yield outcomes in SSA. While climate change-induced changes in SSA’s suitability for maize (Zea mays) production will vary by agro-ecological zone, the overarching trend across agro-ecological zones indicates declining yields^19^. Rainfed production of cereals in SSA is projected to increase due to the use of improved technologies, but potentially attainable yields are likely to be reduced due to changing climatic conditions^20^. Heavier rain will also increase nitrogen leaching, leading to reduced plant uptake and lower yields in nitrogen-deficient -soils^21^. Other studies focused on the effects of temperature have generally found a negative relationship between temperatures and crop productivity^3,22,23^. However, rising temperatures may increase yields in certain regions (i.e., the Ethiopian highlands and the continent’s southern region)^22^. Smallholder farmers in SSA are some of the most vulnerable to the impacts of climate variability and change and identifying opportunities to increase their yields can improve their adaptive capacity^24,25^.

Sub-optimal agronomic factors have led to lower yields compared to the attainable rain-limited crop yield^10,13,26^. This yield gap between actual and attainable crop yields is estimated to be up to 80% for certain crops in SSA countries^27^. This under-productivity is primarily the result of low uptake of improved inputs such as certified seeds and fertilizer^12,15,28,29^, a lack of economic incentives, or services, and insufficient capital for smallholder farmers^25^. Further, improved farm equipment are often designed with large farms in mind, unsuitable for hilly and stony smallholder farms, and uneconomical except through rental schemes or farmers’ associations; as a result, their per capita ownership is also low in SSA^30,42^. Improving physical and economic access to these inputs and services is another vital component for enhancing smallholder productivity.

As evidenced above, a large body of work has sought to understand the relationship between smallholder yields and demographic, agronomic, or climatic factors in isolation. However, little work has evaluated the relative importance of these three sets of factors together in ultimately determining rainfed yield outcomes. Here we explore this knowledge gap by examining the case of rainfed maize in Kenya, where maize cultivation and consumption is widespread, central to farmer livelihoods, and essential for national food security. We leverage detailed, nationally representative farmer survey data for 2010 and 2013 to evaluate the relative importance of demographic, agronomic, and climatic factors in influencing maize yields. By examining the relative effect of these factors, this study aims to identify the factors that offer the greatest opportunity for improvements of rainfed maize yields in Kenya, to understand whether factors under farmers’ control can overcome the effects of climate variability on yields, and to draw broader inferences that are generalizable to similar challenges in rainfed production of other crops in Kenya and in other SSA countries.

## Results

**Figure 1:**
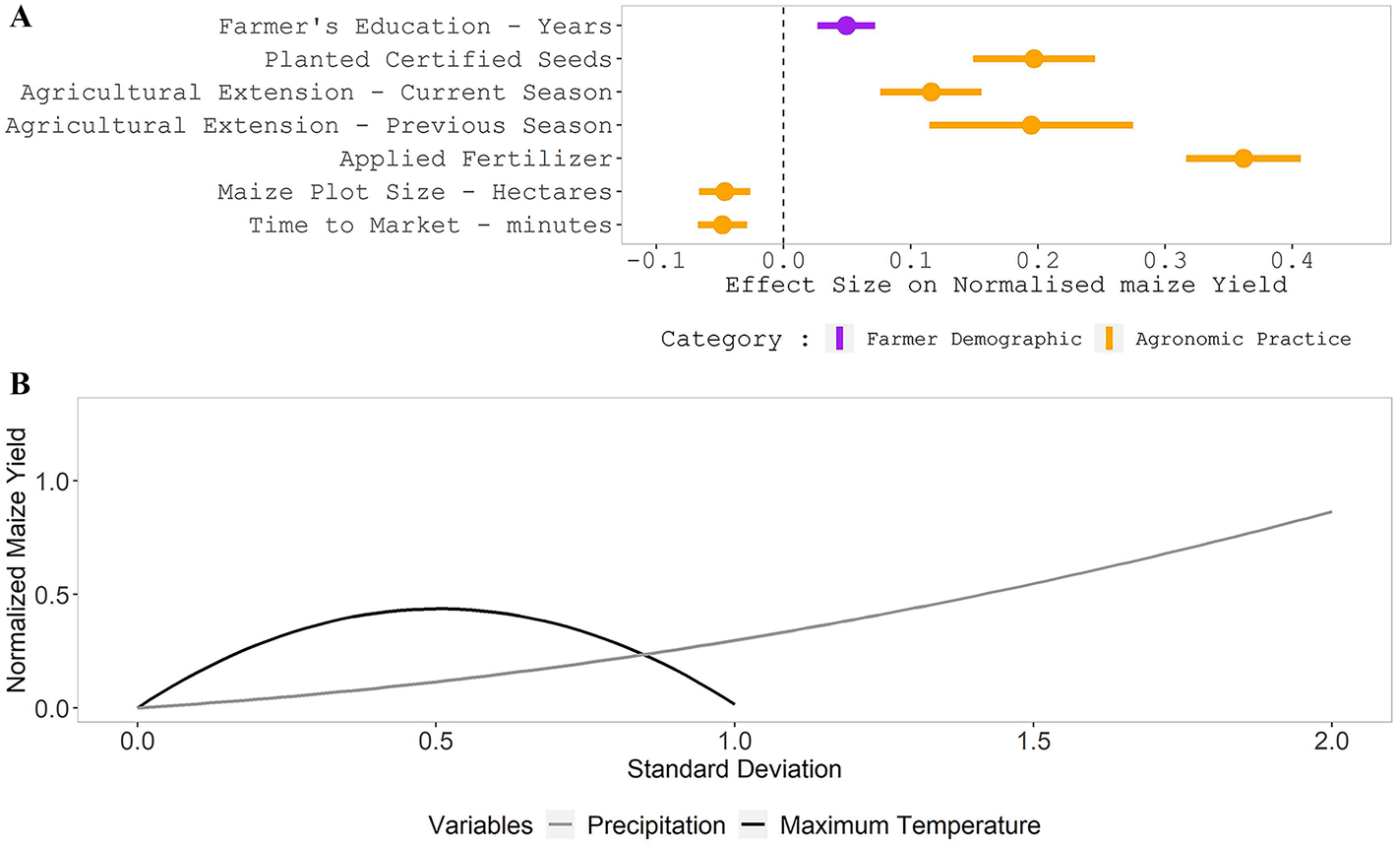
Effect sizes of variables with a significant relationship with normalized maize yield. The bars of similar color across each point (**A**) show the standard error. The effect sizes of climatic variables (**B**) with a significant relationship with normalized maize yield is non-linear because of the squared terms.

We examined the significance and effect-size of each predictor variable within our three groupings: farmer’s demographic information, agronomic factors, and climatic conditions (Fig. 1 and supplementary Table 3).

Model coefficients for the farmer demographic variables had relatively small magnitudes, with farmer education having a significant (*p* = 0.0293) and modest relationship to maize yield. We find that one standard deviation increase in farmer’s education corresponded to a 0.049 unit increase in normalized maize yield. Gender, household size, age, and farming experience showed no statistically significant association with maize yield.

Farmer agronomic factors had the highest number of variables with a significant relationship with normalized maize yield. Farmers who used fertilizer had a 0.362 unit increase in normalized maize yield, while those who planted certified maize seeds were associated with 0.197 units higher productivity than non-certified maize seeds users. For every standard deviation increase in the maize plot size, normalized maize yield was reduced by 0.046—a confirmation of the inverse field size-yield relationship^12,16^. As the normalized time to the nearest market increased by a single unit, the normalized maize yield decreased slightly (− 0.048). At the same time, farmers who had accessed extension services in the current or a previous season had increases of 0.116 and 0.195, respectively, in normalized maize yield units. Access to credit, and distance to (government) extension services did not significantly correlate with normalized maize yield (other factors, such as the use of certified seeds and the application of fertilizer, through which access to credit and distance to extension services may impact maize yields may be influencing their effect-size and statistical significance).

Climatic conditions showed significance in the associations of maximum temperature and normalized maize yield. The linear term of maximum temperature had the largest effect-size of 1.727. However, the squared maximum temperature term had a negative effect of − 1.712, suggesting that the change we observed in normalized maize yield due to maximum temperature has an optimal value beyond which temperature begins to adversely impact yields. One standard deviation increase in annual precipitation linear term had a positive effect of 0.161, and the squared annual precipitation term had a marginally significant coefficient of 0.135.

To verify our results, we created models for each agroecological zone. Overall, the estimated coefficients were consistent in size across all agroecological zones except the climatic variables, and the combined effect of agronomic factors had the most significant impact on normalized maize yield, followed by climatic conditions and, lastly, farmer demographics.

## Discussion

**Figure 2:**
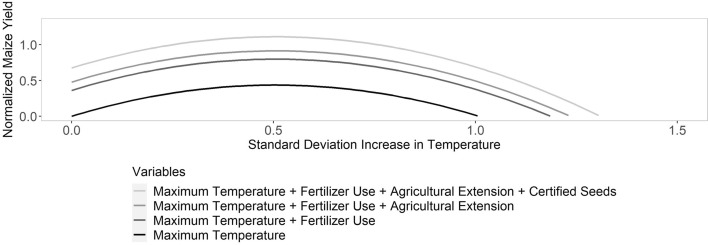
Change in maize yield under selected agronomic factors. Lines show the changes in normalized maize yield associated with a standard deviation increase in maximum temperature in the presence of different combinations of agronomic factors.

Our analysis provides new understanding of the relative importance of demographic, agronomic, and climatic factors in influencing maize yields in Kenya and provides valuable insights into the ways in which these factors may combine to determine yield outcomes for other crops and other countries. We find that agronomic factors have a relatively high influence on yield compared to farmer demographics and offer the greatest opportunity for improvements of rainfed maize yields and counteracting the effects of climatic factors. No single agronomic factor has an effect-size large enough to offset the effects of climatic conditions, and adoption of agronomic factors can be correlated—use of fertilizer and certified seed is an example (Table 1). Thus, a suite of agronomic factors is necessary to improve smallholder yields while adapting to climate change (Fig. 2).

**Table 1:** Correlation matrix of all variables.

	Farmer's age	Farmer's education-years	Farming experience in years	Household size	Farmer's marital status	Farmer's gender	Credit services	Planted certified seeds	Agricultural extension (Current)	Agricultural extension (Previous)	Used fertilizer	Maize plot size	Distance to extension services	Time to market	Maximum temperature	Precipitation
Farmer's Age	1															
Farmer's Education-Years	− 0.361	1														
Farming Experience in Years	0.7173	− 0.3217	1													
Household Size	− 0.0306	0.0352	− 0.0277	1												
Farmer's Marital Status	0.1644	− 0.316	0.1924	− 0.152	1											
Farmer's Gender	− 0.1229	0.3271	− 0.1795	0.1141	− 0.8303	1										
Credit Services	− 0.0214	0.1285	− 0.0294	0.043	− 0.0478	0.0162	1									
Planted Certified Seeds	− 0.0473	0.1811	− 0.0352	0.0085	− 0.1044	0.1021	0.0398	1								
Agricultural Extension (Current)	0.0432	0.0935	0.0122	0.0247	− 0.0486	0.0278	0.1138	0.139	1							
Agricultural Extension (Previous)	0.0414	− 0.0177	0.0662	− 0.0217	− 0.0121	0.0198	− 0.0361	0.0457	0.0859	1						
Used Fertilizer	− 0.0174	0.1842	− 0.0263	− 0.0739	− 0.044	0.0614	0.0436	0.3948	0.0428	− 0.0061	1					
Maize Plot Size	0.0743	0.0686	0.0475	0.1494	− 0.0167	0.0098	0.035	0.0212	0.0737	0.0414	− 0.0823	1				
Distance to Extension services	− 0.0257	− 0.0364	− 0.0452	0.0479	0.0061	0.0029	− 0.0274	− 0.0551	− 0.0035	− 0.0743	− 0.1128	0.059	1			
Time to Market	0.0081	− 0.0402	0.019	0.0054	0.0321	− 0.0334	− 0.0556	− 0.0548	0.0097	0.025	− 0.0855	0.0326	0.0761	1		
Maximum Temperature	− 0.015	− 0.1055	− 0.0549	0.1853	− 0.006	3.00E− 04	− 0.0154	− 0.175	0.0539	− 0.0152	− 0.2809	0.1187	0.0255	0.0546	1	
Precipitation	− 0.0769	0.0465	− 0.0816	0.0245	0.003	− 0.003	− 0.0211	0.1579	− 0.0092	− 0.0424	0.2949	− 0.123	0.0142	− 0.0635	− 0.4148	1

We show that farmers who plant certified seeds, apply fertilizer, or access extension services register higher maize yields; however, due to lack of data, we did not study how the maize yield would vary depending on the type of certified seed planted, how the farmer applied fertilizer, or what extension services they accessed. Efforts aiming to increase yields to feed the growing SSA population and mitigate the effects of climate variability and change should focus on the factors that can most enhance productivity. By comparing the relative effect sizes of each demographic, agronomic, and climatic factor, we show that the mean relative effect-size of agronomic factors is less than that of climatic factors, providing evidence that a suite of agronomic factors is needed to offset the effects of climate variability, consistent with other studies^10,31–35^.

Unlike climatic factors over which farmers have no control, agronomic factors are the set of factors most at agency of SSA farmers. Consequently, efforts to close maize yield gaps—and by extension, the yield gaps for other staple crops—should prioritize targeted improvements in agronomic factors. In particular, our findings provide evidence that faster access to markets, providing extension services, planting certified seeds and applying fertilizer can have an immediate and positive effect on yield outcomes. We also confirm that smallholder farmers with smaller fields tend to be more productive than those with larger fields, as smallholder farmer’s precision in implementing agronomic factors relates inversely to farm size^16,30^. We note though that our findings do not downplay the effect of demographics in potentially improving yields. We find that farmers with higher levels of education tend to have better yield outcomes, which can potentially be attributed to their increased awareness of farming factors and market dynamics^15^. Other farmer demographics, such as gender, have been shown elsewhere to affect yield^17^ despite not having a significant relationship with yield in this study.

While further research is needed to understand interactions of demographic, agronomic, and climatic factors in determining smallholder yields, our study points to multiple opportunities for holistic approaches to improve farmer productivity and to offset adverse impacts of climate change and variability. While our findings are based on rainfed maize cultivation in Kenya, we provide a generalizable methodology and set of results that can be applied to other crops and countries in SSA. Though smallholder options for improved productivity differ based on the crop of interest, government policies, agroecological environments, and climatic conditions, our findings indicate that improved agronomic factors can play an important role in addressing the overarching challenges of population growth, yield gaps, and changing climate.

## Methods

We evaluated the sensitivity of rainfed maize yields in Kenya to demographic, agronomic, and climatic factors using a linear model. We developed the linear model with farmer survey data collected in two calendar years (2010, 2013) covering six agro-ecological zones and thirty-two counties.

### Study area

**Figure 3:**
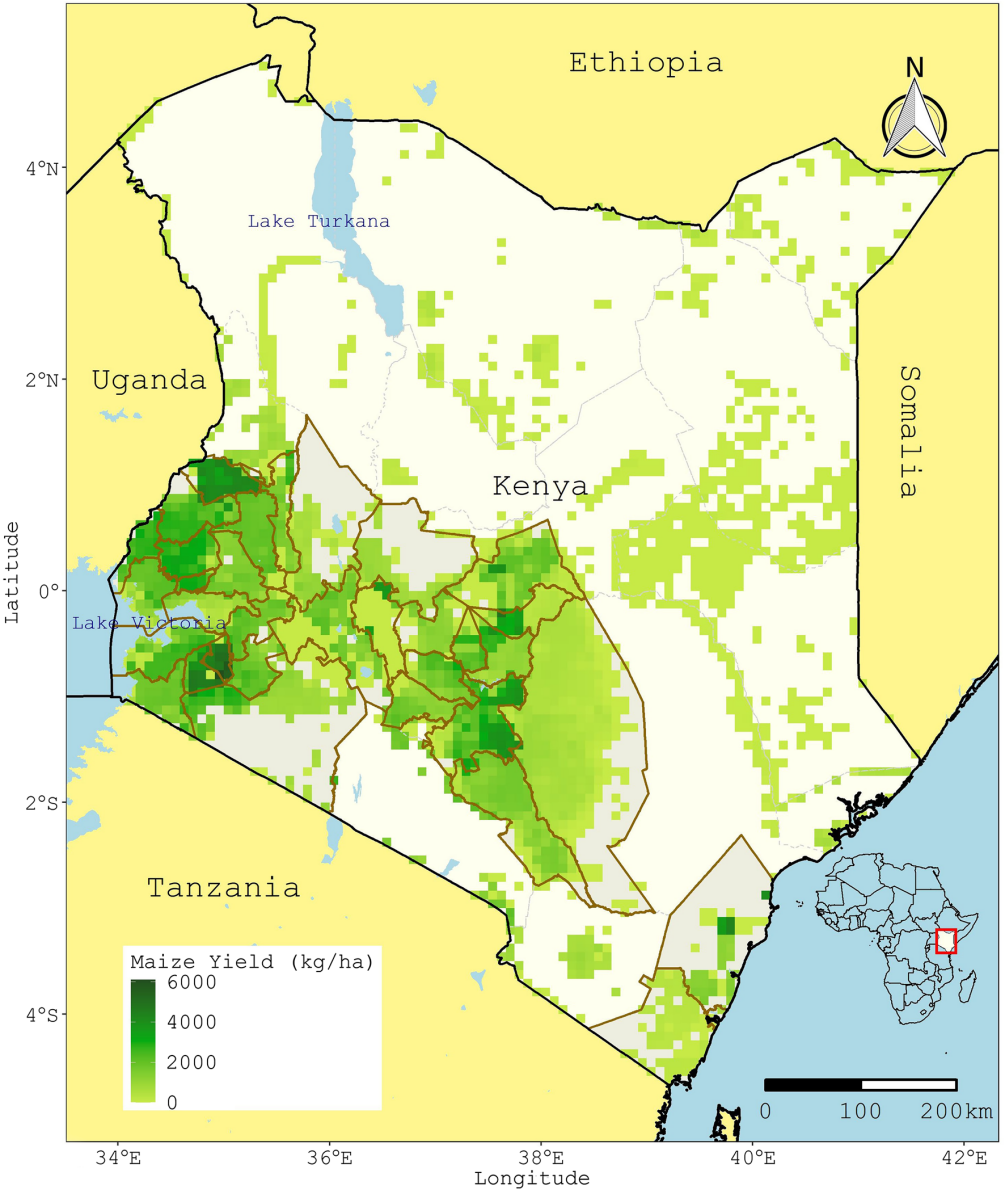
Maize cultivation and survey counties. Gridded maize yield data (the year 2010) came from IFPRI ^36^. The 32 counties covered by the survey are highlighted in light grey with dark brown boundaries, while the other counties have dashed boundaries. Generated using ggplot2 package (version 3.3.5) R version 4.1.2 (Rstudio version 2022.02.0 + 443 in windows 10).

The study focused on the main maize growing areas in Kenya. Kenya is in East Africa and has forty-seven counties (i.e., level-one administrative units). As of 2010, The forty-seven counties were districts under the Kenyan national government. After the March 2013 general elections, the districts' restructuring into counties was completed in line with a devolved system of government outlined in a new Kenya constitution passed in 2010 (for consistency, the word counties is used even for 2010). The study covered thirty-two counties across the southern part of Kenya. In 2010, these thirty-two counties accounted for 91% of total maize production (Fig. 3) and covered 92% of the total area under rainfed maize production^36^.

### Survey: data collection, processing, and cleaning

The International Maize and Wheat Improvement Centre (CIMMYT), in collaboration with the Kenya Agricultural and Livestock Research Organization (KALRO), conducted nationally representative household surveys in the major maize growing areas. The data and the surveys have been previously used to analyze trends in mechanization^37^ and fertilizer use^33^.

The 2010 and 2013 surveys used a two-stage stratified design with six maize agro-ecological zones (AEZs) as strata^31^, census clusters or sublocations as primary sampling units, and maize growing households as secondary sampling units. The first survey, done in 2010, covered 120 sublocations with 1344 households. The second survey, done in 2013, interviewed the same farmers with a 20% replacement with randomly sampled households^34^.

CIMMYT- Nairobi and KALRO surveyed the farmers per the guidelines of the Declaration of Helsinki, and the data was provided for this study by CIMMYT-Nairobi, to the authors, after signing a confidentiality agreement. The farmers provided all information in the survey after being taken through and signing a consent form. We combined data sets from the two surveys and removed personal identifying information and household observations with missing entries. The combined cleaned data for the two years had 17 variables covering 2197 households -1099 in 2010 and 1098 in 2013- in 32 counties (Supplementary Tables 1 and 2).

The 17 variables include one target variable (maize yield in kg ha^−1^) and sixteen independent variables. We organized the independent variables into three broad groups: farmer’s demographic information, agronomic factors, and climatic conditions. There were six variables on farmer demographics: gender, marital status, age, size of household, years in farming, and years of education; eight variables on agronomic factors: area under maize, use of certified maize seeds, use of fertilizer, access to extension services (in current and previous seasons), distance to the nearest extension service center, access to credit services, and, time to the nearest market; and two on climatic conditions (i.e., variables largely out of a farmer’s control): maximum temperature (at 2 m height), and total precipitation in the growing season. The original dataset also included a minimum temperature variable, which we excluded from the analysis, as it provided near-identical information to maximum temperature in explaining yield variability. We mapped the spatial distributions of the independent variables to identify spatial trends in the data (Supplementary Figs. 1–30).

We transformed each independent variable to the appropriate data structure before using them in the linear model. First, we added the squared terms of the climate variables (maximum temperature and precipitation) as new variables because of the non-linear relationship between climate and yield. Then, we set all binary variables (Table 2) as dummy variables with their absence as the reference value, set marital status as a categorical variable of three levels, and normalized the numeric variables. We normalized numeric variables to remove the effects of measurement units in two steps: subtracting the mean from each observation and dividing this difference by the standard deviation. Before normalizing, the climate variables were divided into groups based on the agroecological zones, and each group normalized separately. Lastly, we calculated the variance inflation factor (VIF) to eliminate colinear independent variables based on a standard VIF threshold of five. The VIF value of all variables was less than the threshold, so we did not remove any.

**Table 2:** Summary of study data characteristics after standardization.

Variable	Category	Unit	Minimum	Maximum	Median	SD (Standard Deviation)
Farmer’s age	Farmers' demographics	Years	− 2.31	3.17	− 0.02	1
Farmer’s education	Farmers' demographics	Years of schooling	− 1.68	4.07	0	1
Farmer’s experience	Farmers' demographics	Years	− 1.73	3.52	− 0.12	1
Farmer’s household size	Farmers' demographics	Persons	− 2.01	5.51	− 0.13	1
Farmer’s Relationship status	Farmers' demographics	(Nominal scale: 0 to 3)	0	3	0	0.81
Farmer’s gender	Farmers' demographics	(Binary: 0 for female and 1 for male)	0	1	1	0.4
The farmer has access to credit	Farmers' agronomic factors	(Binary: 0 for false and 1 for true)	0	1	1	0.5
The farmer planted certified seeds	Farmers' agronomic factors	(Binary: 0 for false and 1 for true)	0	1	1	0.44
Agricultural extension-current season	Farmers' agronomic factors	(Binary: 0 for false and 1 for true)	0	1	0	0.5
Agricultural extension-previous season	Farmers' agronomic factors	(Binary: 0 for false and 1 for true)	0	1	1	0.24
The farmer used fertilizer	Farmers' agronomic factors	(Binary: 0 for False and 1 for True)	0	1	1	0.5
Size of plot under maize	Farmers' agronomic factors	Hectares	− 0.68	20.68	− 0.29	1
Distance from the farm to the extension services	Farmers' agronomic factors	Kilometers	− 0.83	12.34	− 0.29	1
Time of travel from the farm to the market	Farmers' agronomic factors	Minutes	− 0.8	17.59	− 0.19	1
Maximum temperature in the maize farm’s location (growing season)	Farmers' climatic conditions	Degree Celsius	− 3.1	2.04	0.24	1
Total precipitation (growing season)	Farmers' climatic conditions	Millimeters	− 1.23	5.96	− 0.05	1

### Linear model

Using the normalized data, we developed a linear model to examine how sensitive maize yield (kg ha^−1^) was to farmers’ demographic information, agronomic factors, and climatic conditions following^38^ and^39^. We computed the linear model and performed the data normalization and VIF calculation steps described in  "Survey: data collection, processing, and cleaning" section in R using functions from stats, fmsb, and R base packages. We organized the developed functions as a new R package and anonymized the data used in this study before including it in the R package, which we called “yieldest.”The R package is publicly available on GitHub^40^ and Harvard Dataverse^41^. Using normalized maize yield as the target variable and the normalized independent variables, we could compare the magnitude of coefficients (i.e., effect size) in the linear model to evaluate the relative influence of corresponding predictor variables in determining rainfed maize yields.

### Equipment and settings

We generated all figures in this manuscript using ggplot2 package R version 4.1.2 (Rstudio version 2022.02.0 + 443 in windows 10) with data from our results and gridded maize yield data (the year 2010) from^35^.

## Supplementary Information

Supplementary Information.

## Data Availability

The datasets generated and/or analyzed during the current study are available in the Harvard Dataverse repository, https://doi.org/10.7910/DVN/UIWQQH. Contact corresponding author for data requests.
