# Supplementary material for: A suite of agronomic factors can offset the effects of climate variability on rainfed maize production in Kenya
Source: Sci Rep. 2022 Oct 3;12:16043. doi: 10.1038/s41598-022-19286-2 (PMC9529968; doi:10.1038/s41598-022-19286-2)
Supplement: Supplementary file 1 — Supplementary Information. [file 41598_2022_19286_MOESM1_ESM.docx]

**Supplementary Material**

**Supplementary Table 1. Distribution of Respondents in 2010.**

| Parameter | Category | unit | minimum | maximum | mean | median | sd | No of 0's | No of 1's |
| --- | --- | --- | --- | --- | --- | --- | --- | --- | --- |
| Maize Yield |  | KG/ Hectare | 0 | 6666.67 | 1048.13 | 740.74 | 979.92 |  |  |
| Farmer’s age | Farmers' demographics | Years | 20 | 98 | 52.49 | 52 | 14.77 |  |  |
| Farmer’s education | Farmers' demographics | Years of schooling | 0 | 24 | 6.58 | 7 | 4.16 |  |  |
| Farmer’s experience | Farmers' demographics | Years | 0 | 74 | 24.77 | 23 | 15.08 |  |  |
| Farmer’s household size | Farmers' demographics | Persons | 1 | 18 | 6.23 | 6 | 2.72 |  |  |
| Farmer’s Relationship status | Farmers' demographics | (Nominal scale: 0 to 3) | 0 | 3 | 0.42 | 0 | 0.83 |  |  |
| Farmer’s gender | Farmers' demographics | (Binary: 0 for female and 1 for male) | 0 | 1 | 0.8 | 1 | 0.4 | 220 | 879 |
| The farmer has access to credit | Farmers' agronomic practices | (Binary: 0 for false and 1 for true) | 0 | 1 | 0.49 | 0 | 0.5 | 564 | 535 |
| The farmer planted certified seeds | Farmers' agronomic practices | (Binary: 0 for false and 1 for true) | 0 | 1 | 0.67 | 1 | 0.47 | 361 | 738 |
| Agricultural extension - current season | Farmers' agronomic practices | (Binary: 0 for false and 1 for true) | 0 | 1 | 0.06 | 0 | 0.23 | 1036 | 63 |
| Agricultural extension - previous season | Farmers' agronomic practices | (Binary: 0 for false and 1 for true) | 0 | 1 | 0.94 | 1 | 0.24 | 69 | 1030 |
| The farmer used fertilizer | Farmers' agronomic practices | (Binary: 0 for False and 1 for True) | 0 | 1 | 0.53 | 1 | 0.5 | 519 | 580 |
| Size of the total area under maize | Farmers' agronomic practices | Hectares | 0.01 | 28.35 | 1.26 | 0.81 | 1.65 |  |  |
| Distance from farm to extension services | Farmers' agronomic practices | Kilometers | 0 | 120 | 7.6 | 5 | 9.11 |  |  |
| Time of travel from farm to the market | Farmers' agronomic practices | Minutes | 0 | 604 | 26.23 | 20 | 32.85 |  |  |
| Maximum annual temperature in the maize farm’s location (growing season) | Farmers' climatic conditions | Degree Celsius | 21.83 | 36.38 | 30.53 | 30.86 | 2.9 |  |  |
| Total precipitation (growing season) | Farmers' climatic conditions | Millimeters | 370.68 | 7582.68 | 1626.51 | 1517.56 | 1046.47 |  |  |

**Supplementary Table 2. Distribution of Respondents in 2013.**

| Parameter | Category | unit | minimum | maximum | mean | median | sd | No of 0's | No of 1's |
| --- | --- | --- | --- | --- | --- | --- | --- | --- | --- |
| Maize Yield |  | KG/ Hectare | 0 | 10814.81 | 1189.19 | 733.33 | 1430 |  |  |
| Farmer’s age | Farmers' demographics | Years | 23 | 99 | 54.15 | 54 | 14 |  |  |
| Farmer’s education | Farmers' demographics | Years of schooling | 0 | 21 | 7.43 | 8 | 4.15 |  |  |
| Farmer’s experience | Farmers' demographics | Years | 2 | 75 | 24.54 | 22 | 13.46 |  |  |
| Farmer’s household size | Farmers' demographics | Persons | 1 | 21 | 6.46 | 6 | 2.59 |  |  |
| Farmer’s Relationship status | Farmers' demographics | (Nominal scale: 0 to 3) | 0 | 3 | 0.39 | 0 | 0.79 |  |  |
| Farmer’s gender | Farmers' demographics | (Binary: 0 for female and 1 for male) | 0 | 1 | 0.8 | 1 | 0.4 | 222 | 876 |
| The farmer has access to credit | Farmers' agronomic practices | (Binary: 0 for false and 1 for true) | 0 | 1 | 0.64 | 1 | 0.48 | 400 | 698 |
| The farmer planted certified seeds | Farmers' agronomic practices | (Binary: 0 for false and 1 for true) | 0 | 1 | 0.79 | 1 | 0.41 | 231 | 867 |
| Agricultural extension - current season | Farmers' agronomic practices | (Binary: 0 for false and 1 for true) | 0 | 1 | 0.85 | 1 | 0.35 | 161 | 937 |
| Agricultural extension - previous season | Farmers' agronomic practices | (Binary: 0 for false and 1 for true) | 0 | 1 | 0.94 | 1 | 0.24 | 69 | 1029 |
| The farmer used fertilizer | Farmers' agronomic practices | (Binary: 0 for False and 1 for True) | 0 | 1 | 0.59 | 1 | 0.49 | 446 | 652 |
| Size of the total area under maize | Farmers' agronomic practices | Hectares | 0.05 | 42.93 | 1.52 | 0.81 | 2.31 |  |  |
| Distance from farm to extension services | Farmers' agronomic practices | Kilometers | 0 | 120 | 7.6 | 5 | 9.11 |  |  |
| Time of travel from farm to the market | Farmers' agronomic practices | Minutes | 0 | 604 | 26.24 | 20 | 32.86 |  |  |
| Maximum annual temperature in the maize farm’s location (growing season) | Farmers' climatic conditions | Degree Celsius | 22.64 | 36.91 | 31.33 | 31.98 | 2.9 |  |  |
| Total precipitation (growing season) | Farmers' climatic conditions | Millimeters | 366.12 | 6511.21 | 1580.5 | 1566.67 | 958.15 |  |  |

**Supplementary Table 3. Coefficients and significance of the linear model variables.** *: p-value between 0.01 and 0.05; **: p-value between 0.001 and 0.01; ***: p-value<0.001

| **Parameter**  (Intercept)  Farmer’s age  Farmer’s education  Farmer’s experience  Farmer’s household size  Farmer’s Relationship status (1)  Farmer’s Relationship status (2)  Farmer’s Relationship status (3)  Farmer’s gender (male)  The farmer has access to credit (true)  The farmer planted certified seeds (true)  Agricultural extension - current season  Agricultural extension - previous season  The farmer used fertilizer  Size of the total area under maize  Distance from farm to extension services  Time of travel from farm to the market  Maximum annual temperature in the maize farm’s location (growing season)  Total precipitation (growing season)  Squared maximum annual temperature in maize farm’s location (growing season)  Squared Total precipitation (growing season) | **Category**  Farmers' demographics  Farmers' demographics  Farmers' demographics  Farmers' demographics  Farmers' demographics  Farmers' demographics  Farmers' demographics  Farmers' demographics  Farmers' agronomic practices  Farmers' agronomic practices  Farmers' agronomic practices  Farmers' agronomic practices  Farmers' agronomic practices  Farmers' agronomic practices  Farmers' agronomic practices  Farmers' agronomic practices  Farmers' climatic conditions  Farmers' climatic conditions  Farmers' climatic conditions  Farmers' climatic conditions | **Estimate**  -0.5678  -0.0362  0.0492  -0.0086  0.0205  0.1009  -0.0289  -0.0032  -0.0275  0.0205  0.1969  0.1158  0.1946  0.3616  -0.0464  0.0193  -0.0481  1.7273  0.1612  -1.7123  0.1355 | **Std. Error**  0.1239  0.0284  0.0225  0.028  0.0201  0.166  0.0925  0.2019  0.09  0.0393  0.048  0.0398  0.0801  0.0449  0.0202  0.0194  0.0193  0.3005  0.0566  0.3001  0.0548 | **P-value**  0  0.2026  0.0293  0.7586  0.3068  0.5433  0.7548  0.9875  0.7604  0.6015  0  0.0037  0.0152  0  0.0218  0.3216  0.0129  0  0.0045  0  0.0136 | **Significance**  ***  *  ***  **  *  ***  *  *  ***  **  ***  * |
| --- | --- | --- | --- | --- | --- |


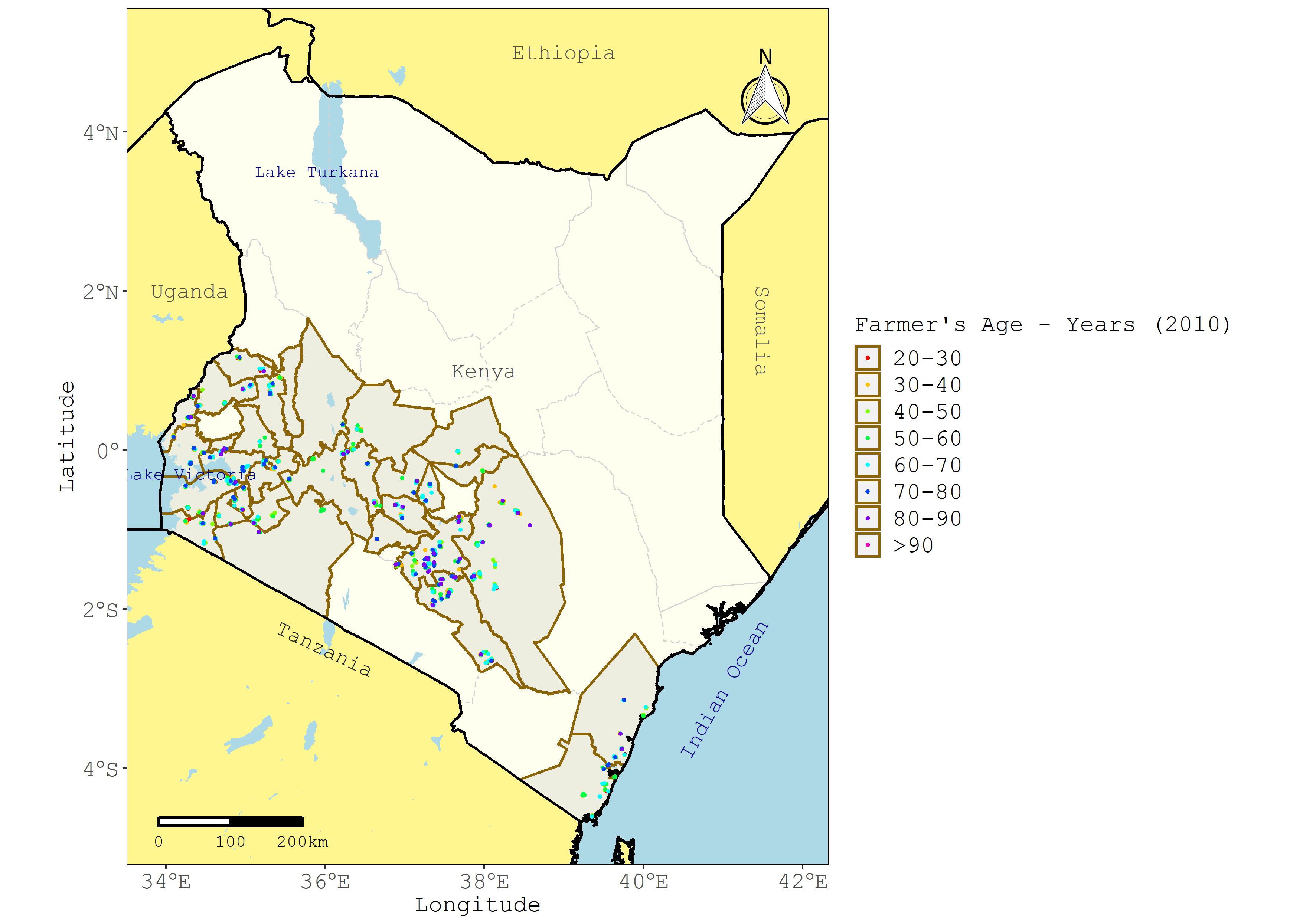


Supplementary Figure 1. Spatial distribution of farmers’ age in 2010. Generated using ggplot2 package (version 3.3.5) R version 4.1.2 (Rstudio version 2022.02.0+443 in windows 10).


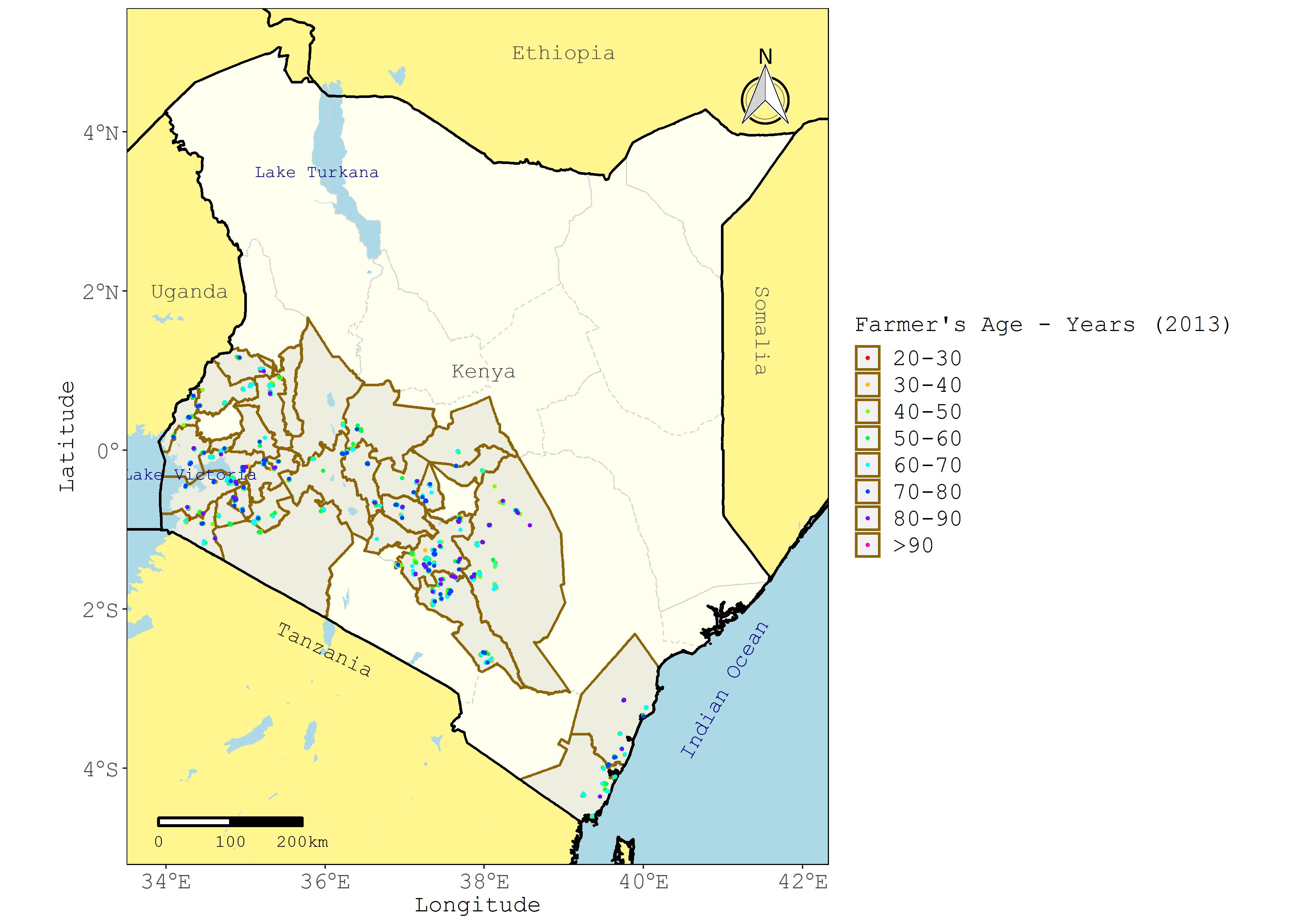


Supplementary Figure 2. Spatial distribution of farmers’ age in 2013. Generated using ggplot2 package (version 3.3.5) R version 4.1.2 (Rstudio version 2022.02.0+443 in windows 10).


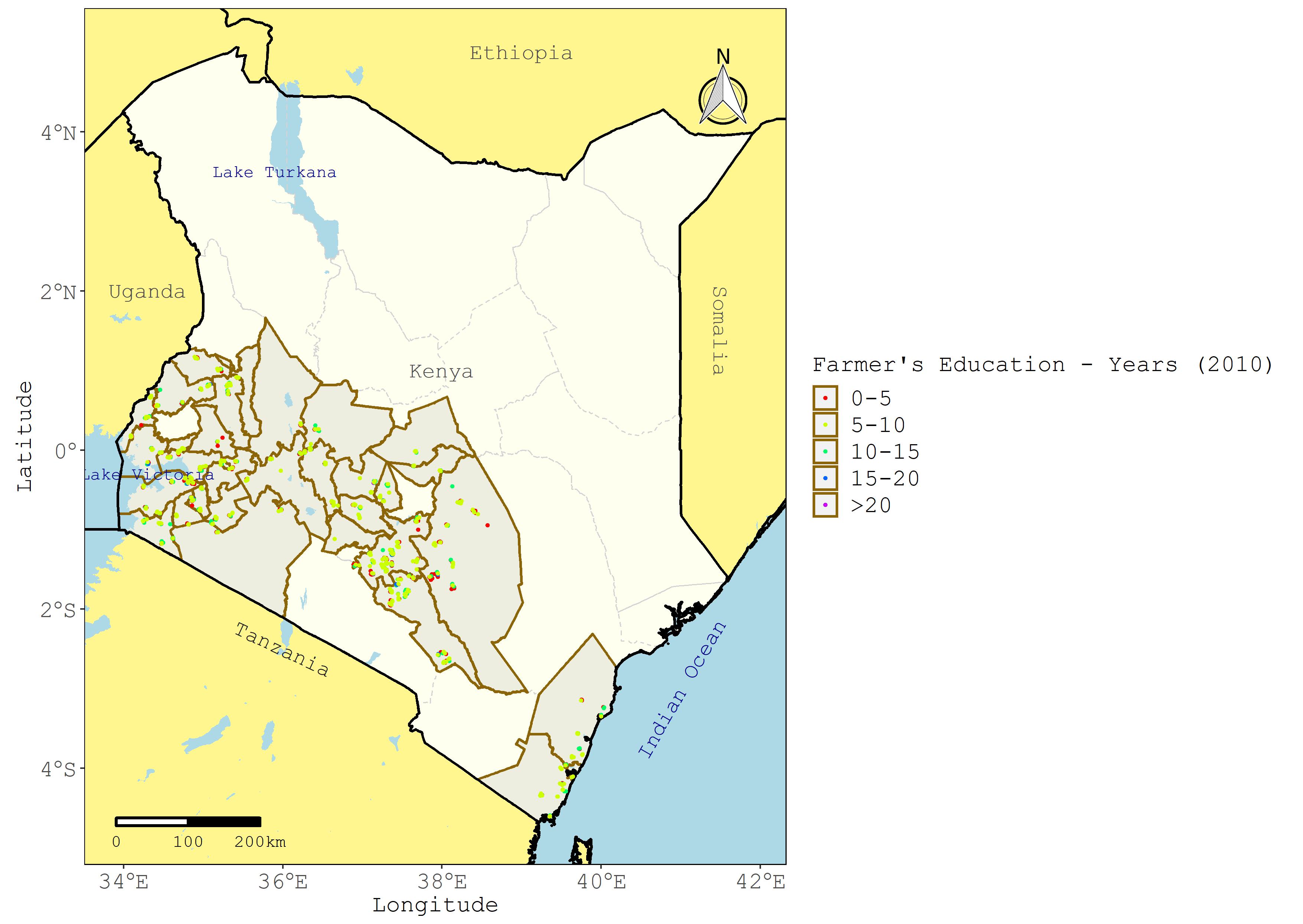


Supplementary Figure 3. Distribution of farmers’ Education level in 2010. Generated using ggplot2 package (version 3.3.5) R version 4.1.2 (Rstudio version 2022.02.0+443 in windows 10).


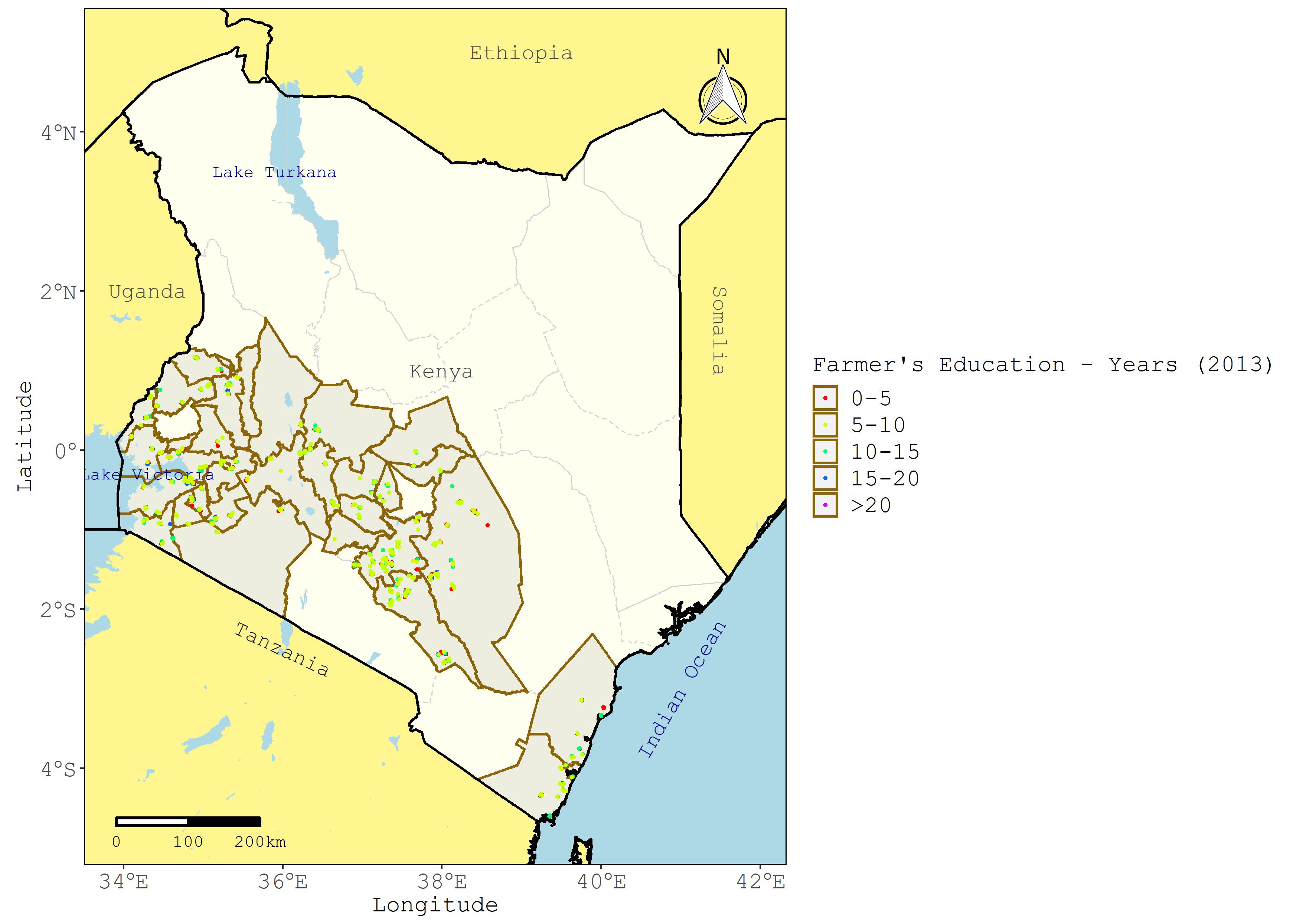


Supplementary Figure 4. Distribution of farmers’ Education level in 2013. Generated using ggplot2 package (version 3.3.5) R version 4.1.2 (Rstudio version 2022.02.0+443 in windows 10).


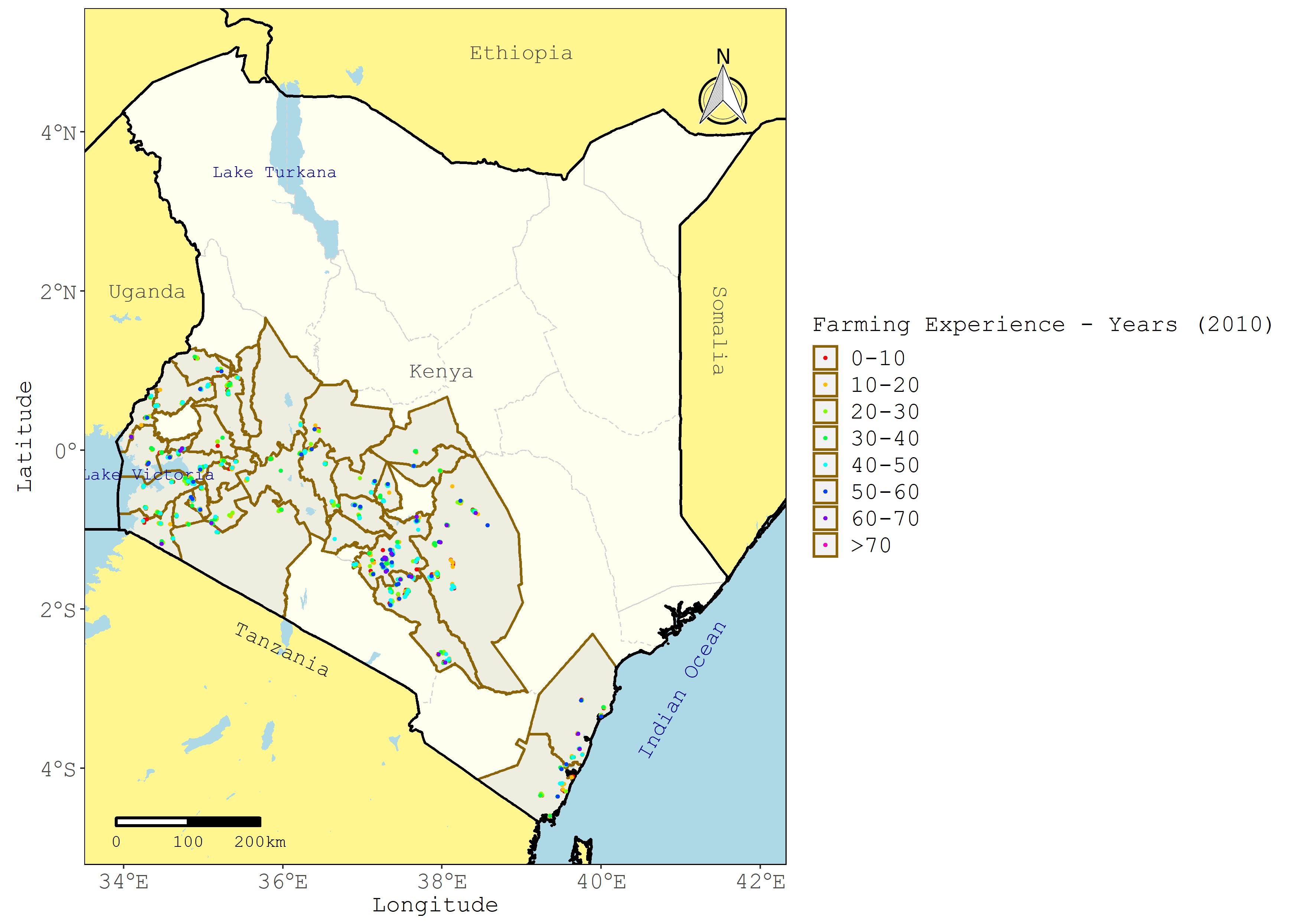


Supplementary Figure 5. Spatial distribution of farming experience in 2010. Generated using ggplot2 package (version 3.3.5) R version 4.1.2 (Rstudio version 2022.02.0+443 in windows 10).


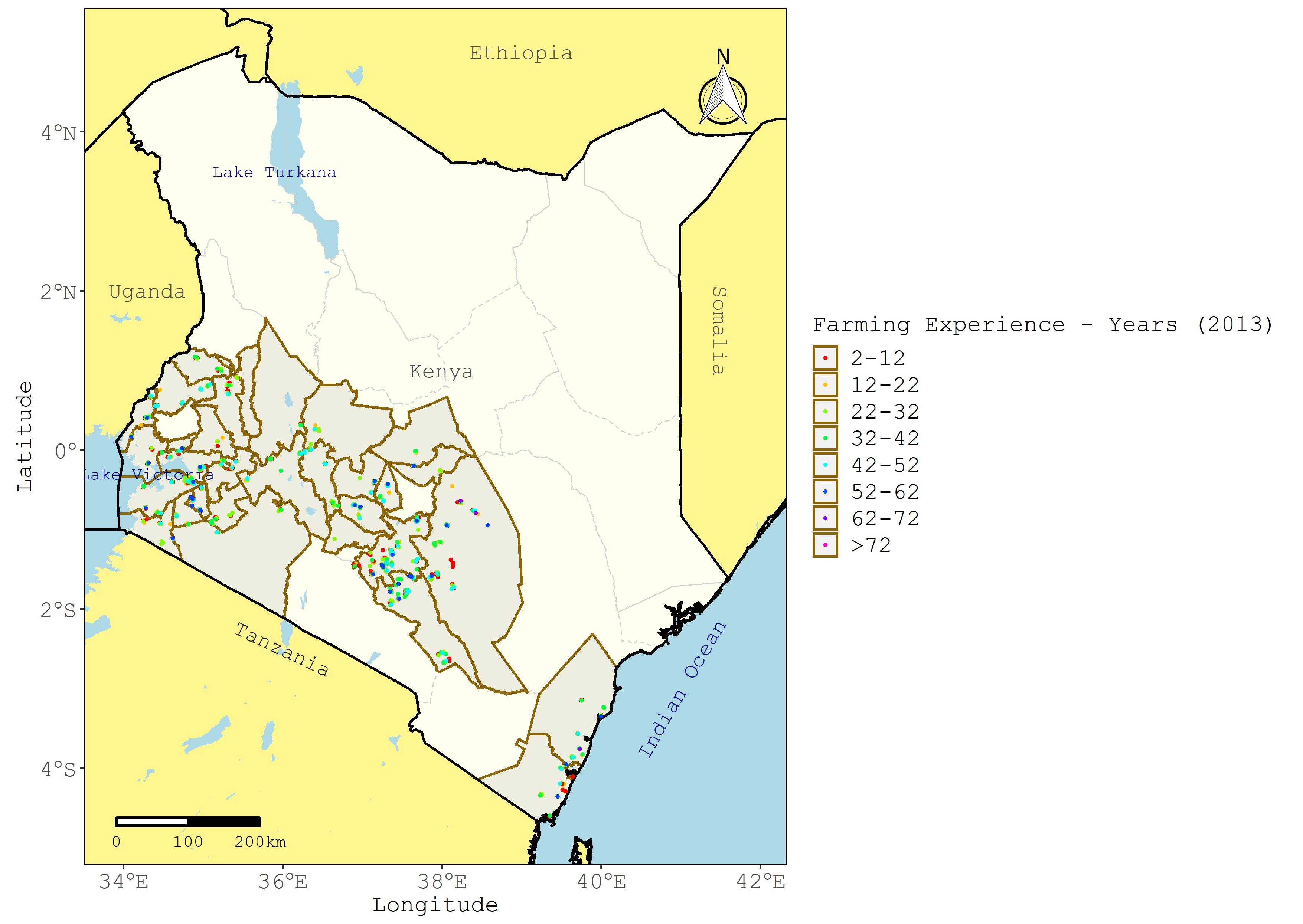


Supplementary Figure 6. Spatial distribution of farming experience in 2013. Generated using ggplot2 package (version 3.3.5) R version 4.1.2 (Rstudio version 2022.02.0+443 in windows 10).


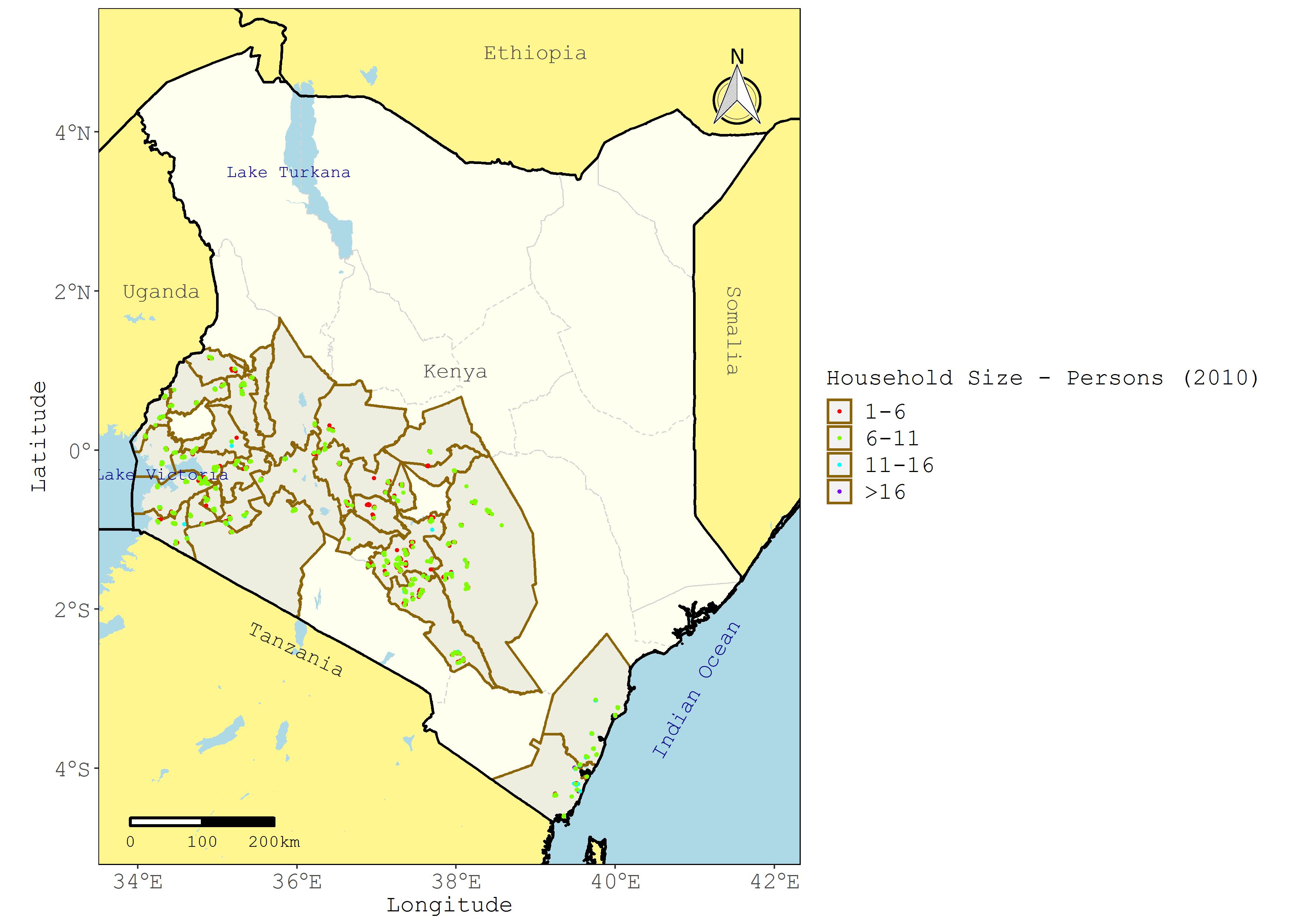


Supplementary Figure 7. Spatial distribution of household sizes in 2010. Generated using ggplot2 package (version 3.3.5) R version 4.1.2 (Rstudio version 2022.02.0+443 in windows 10).


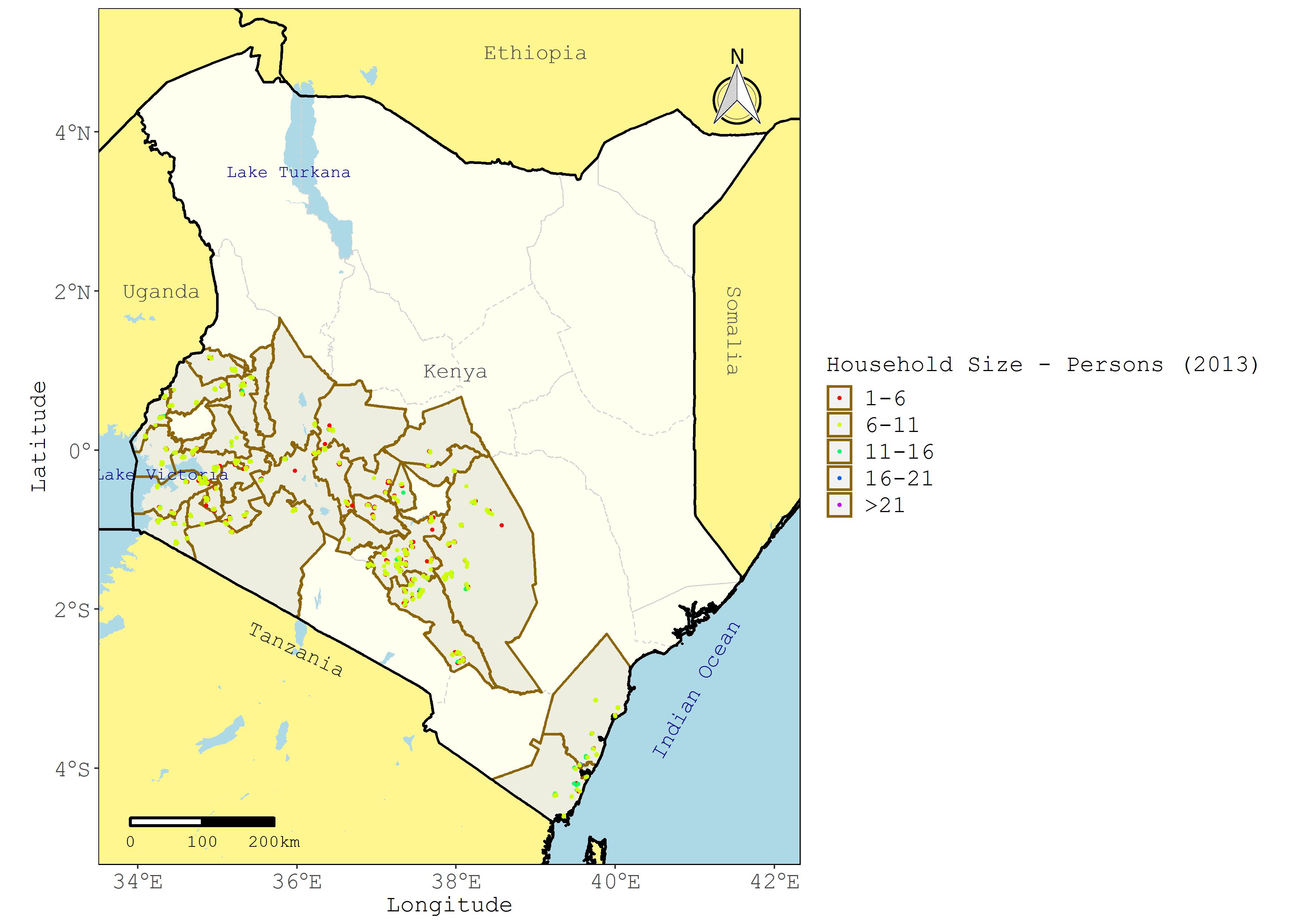


Supplementary Figure 8. Spatial distribution of household sizes in 2013. Generated using ggplot2 package (version 3.3.5) R version 4.1.2 (Rstudio version 2022.02.0+443 in windows 10).


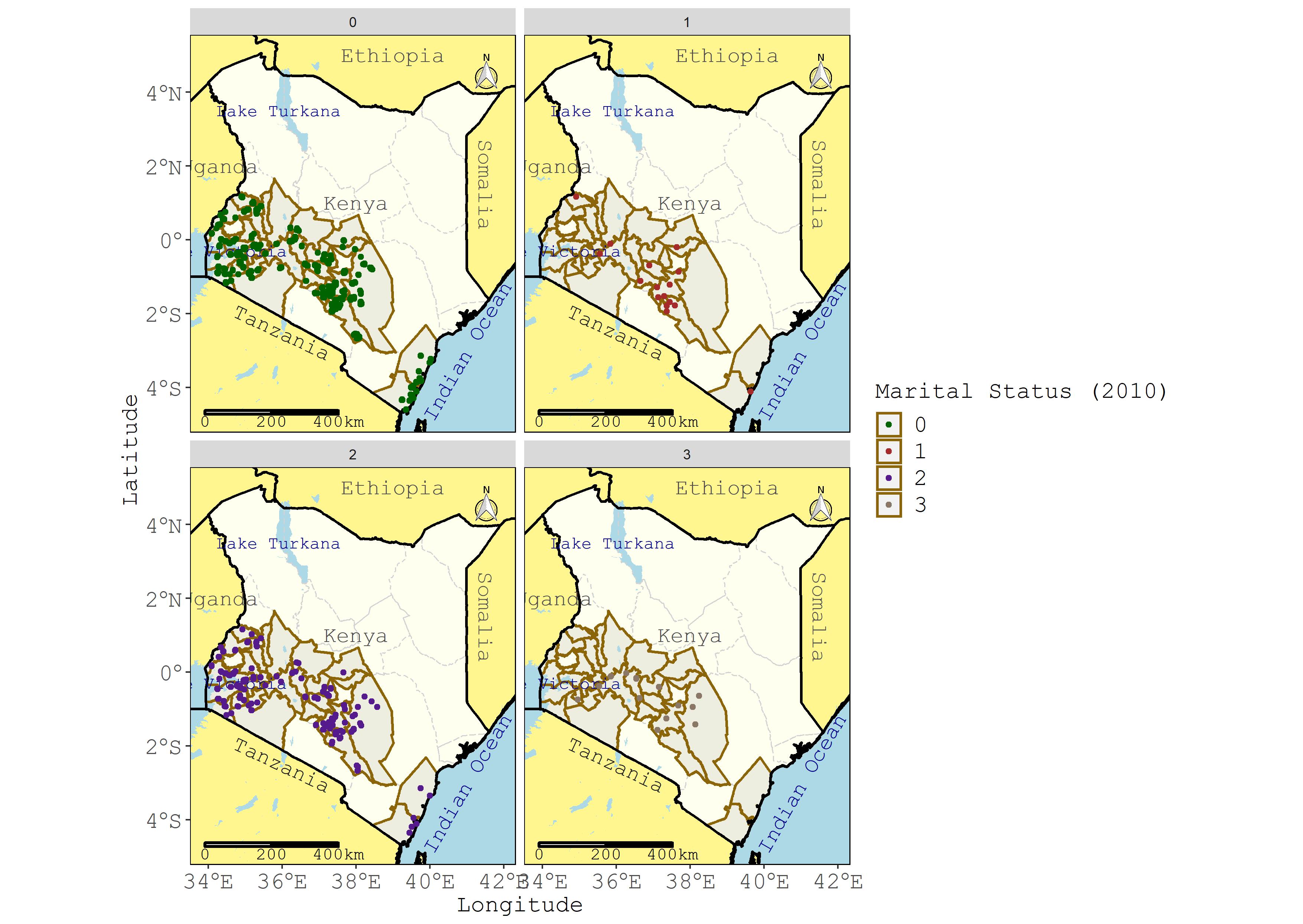


Supplementary Figure 9. Spatial distribution on farmers by marital status in 2010. The marital status was considered personally identifiable information and consequently coded for privacy. Generated using ggplot2 package (version 3.3.5) R version 4.1.2 (Rstudio version 2022.02.0+443 in windows 10).


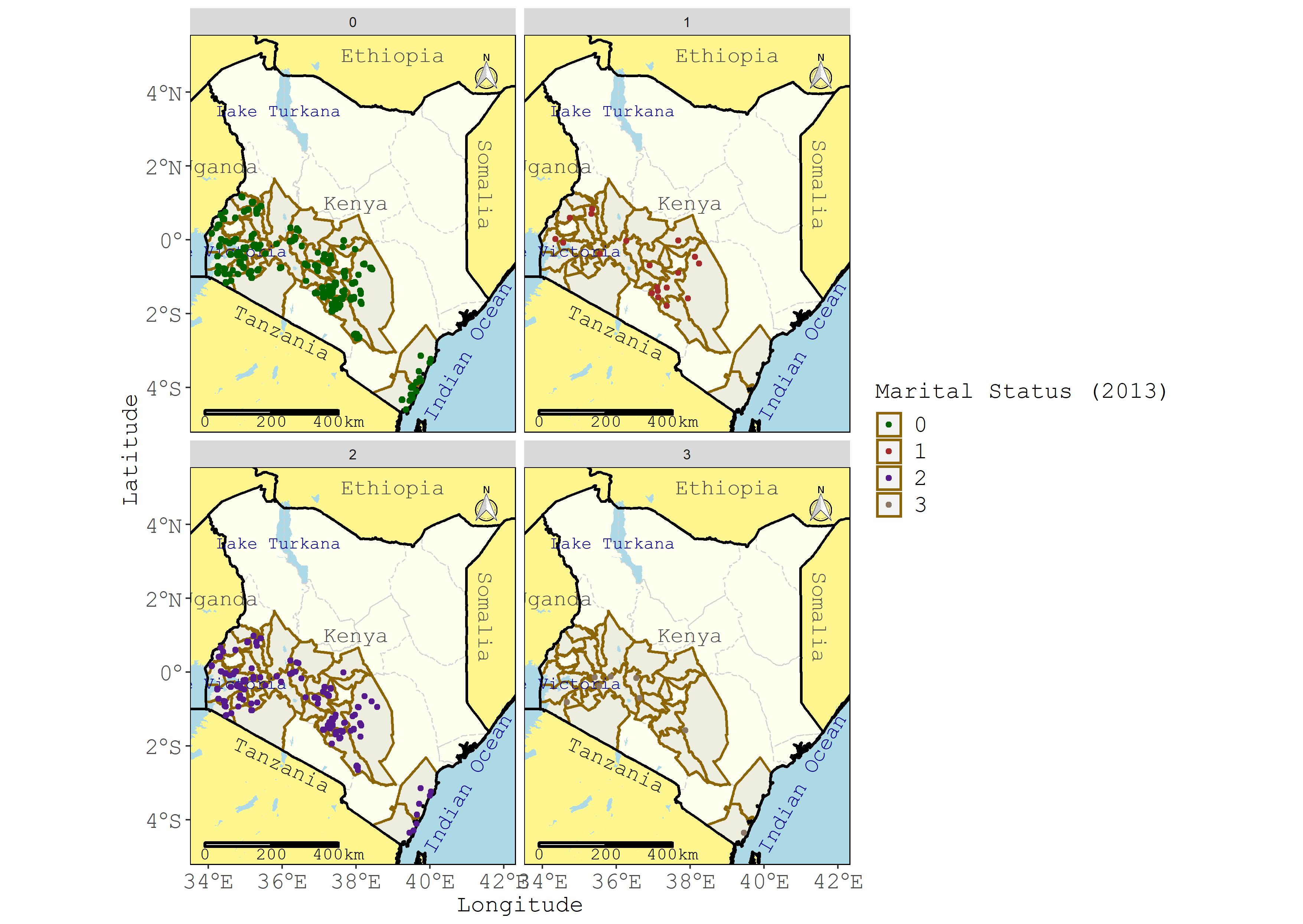


Supplementary Figure 10. Spatial distribution on farmers by marital status in 2013. The marital status was considered personally identifiable information and consequently coded for privacy. Generated using ggplot2 package (version 3.3.5) R version 4.1.2 (Rstudio version 2022.02.0+443 in windows 10).


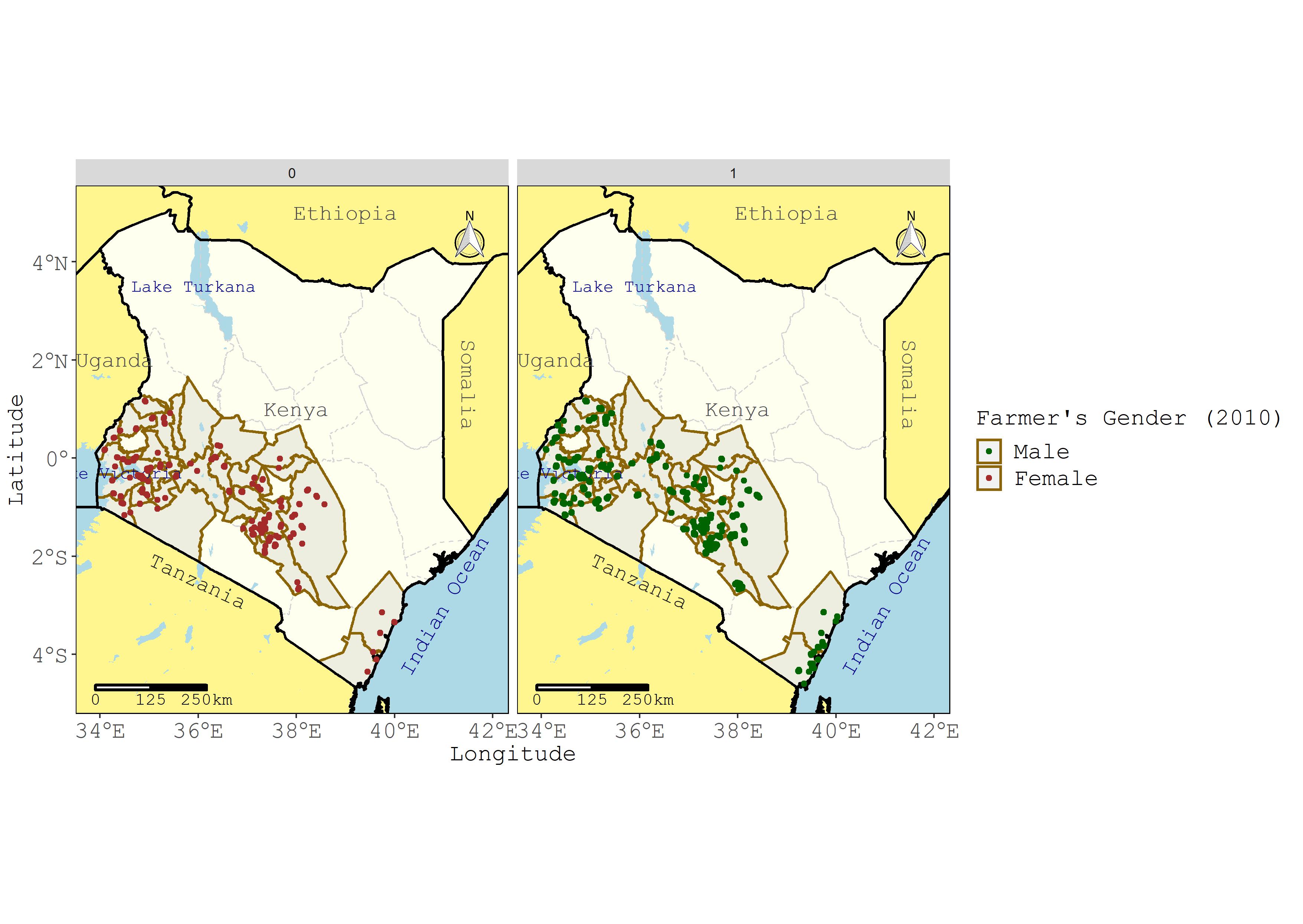


Supplementary Figure 11. Spatial distribution of male and female farmers in 2010. Generated using ggplot2 package (version 3.3.5) R version 4.1.2 (Rstudio version 2022.02.0+443 in windows 10).


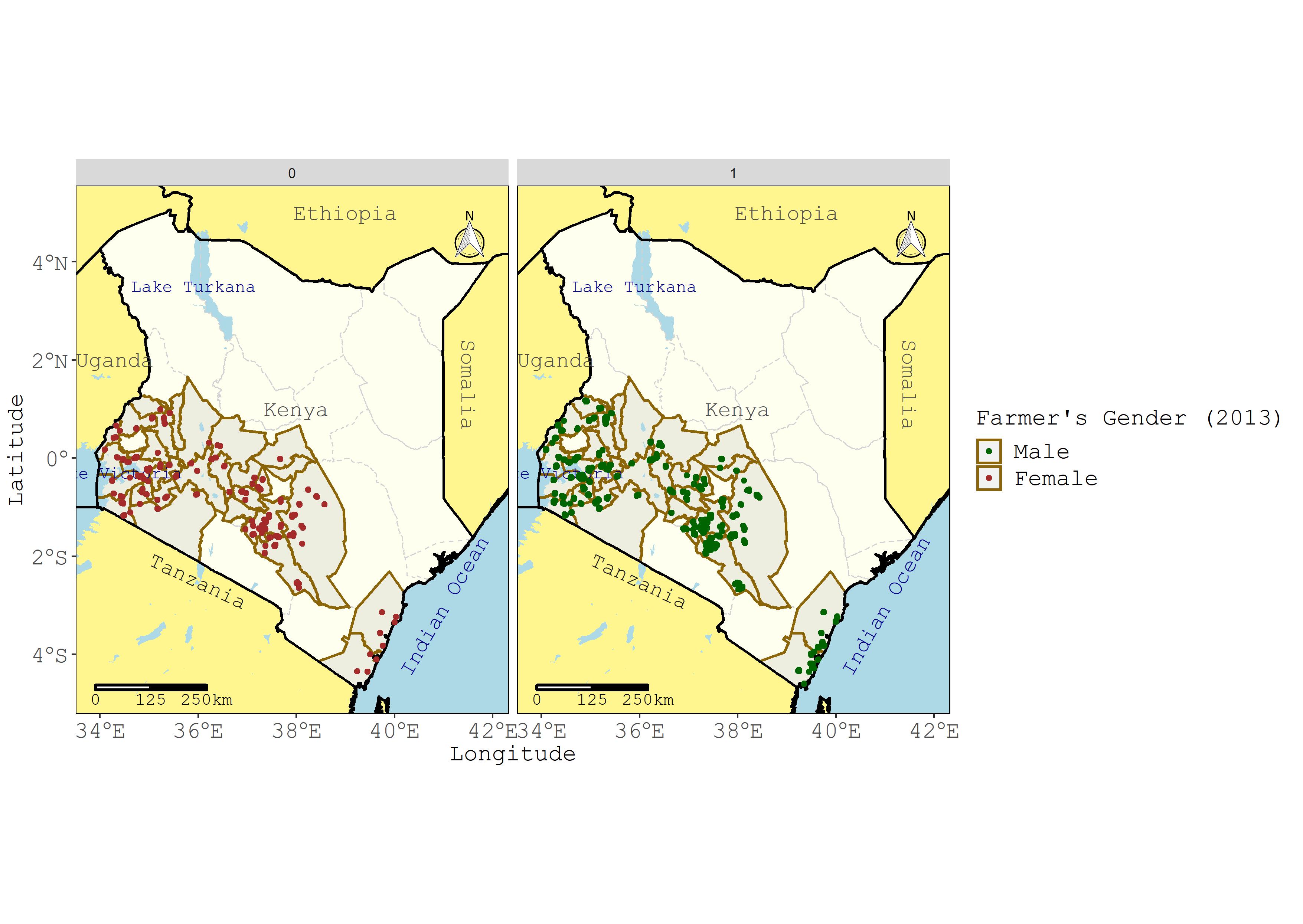


Supplementary Figure 12. Spatial distribution of male and female farmers in 2013. Generated using ggplot2 package (version 3.3.5) R version 4.1.2 (Rstudio version 2022.02.0+443 in windows 10).


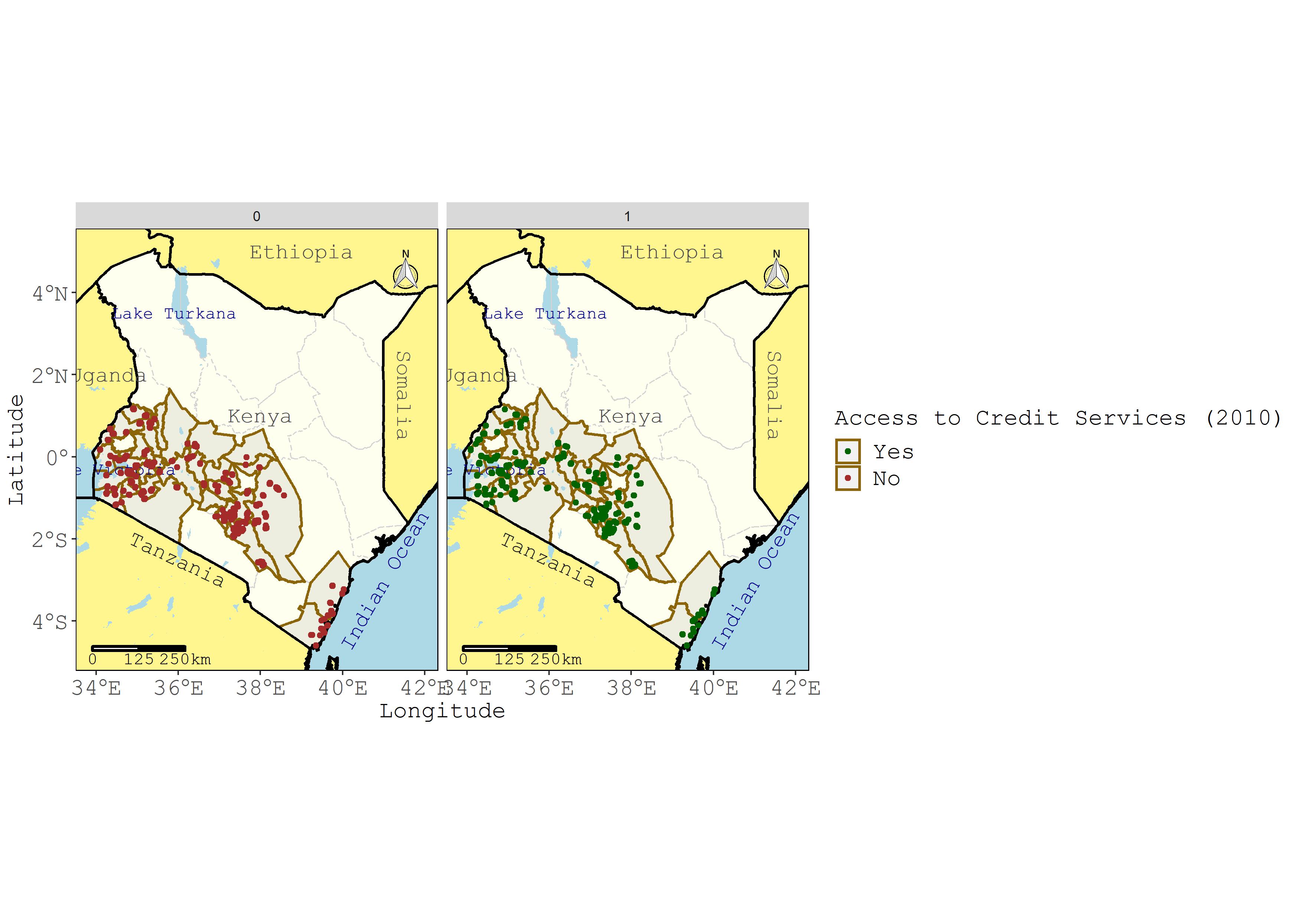


Supplementary Figure 13. Location of farmers who accessed credit services or did not in 2010. The values 1 and 0 (title) are inputs to “Access to Credit Services” dummy variable for either case respectively. Generated using ggplot2 package (version 3.3.5) R version 4.1.2 (Rstudio version 2022.02.0+443 in windows 10).


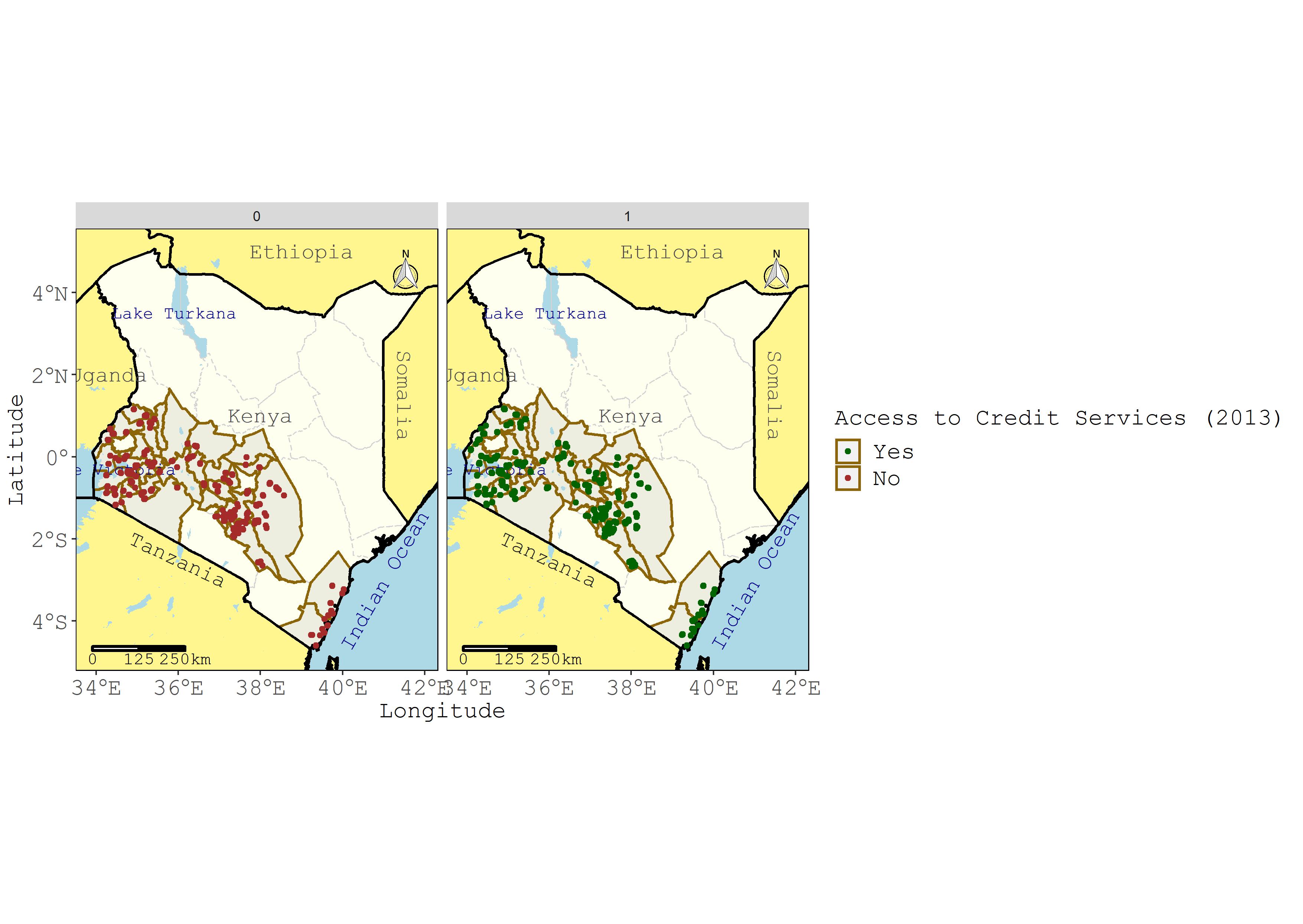


Supplementary Figure 14. Location of farmers who accessed credit services or did not in 2013. The values 1 and 0 (title) are inputs to “Access to Credit Services” dummy variable for either case respectively. Generated using ggplot2 package (version 3.3.5) R version 4.1.2 (Rstudio version 2022.02.0+443 in windows 10).


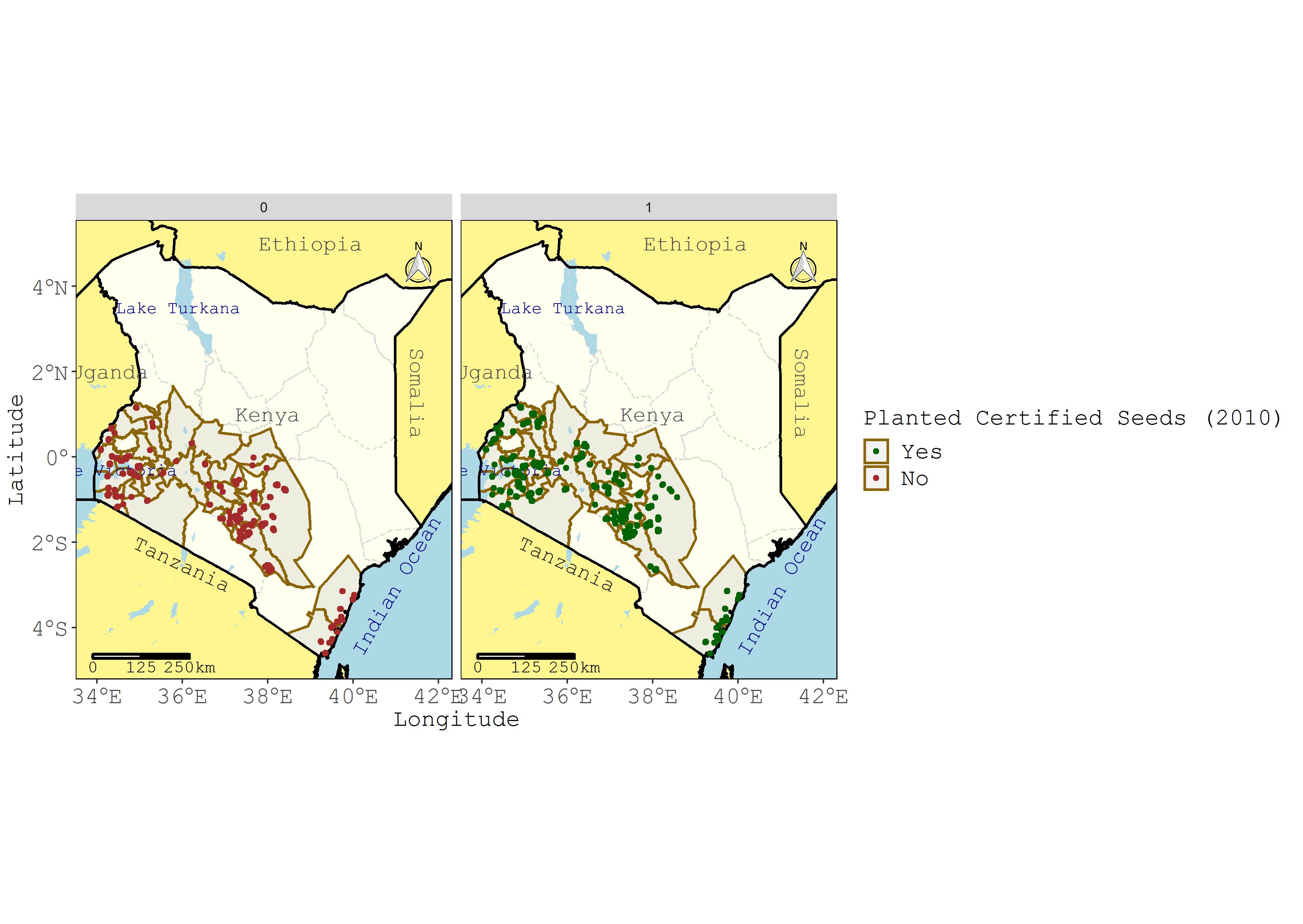


Supplementary Figure 15. Location of farmers who planted certified seeds or did not in 2010. The values 1 and 0 (title) are inputs to “Planted Certified Seeds” dummy variable for either case respectively. Generated using ggplot2 package (version 3.3.5) R version 4.1.2 (Rstudio version 2022.02.0+443 in windows 10).


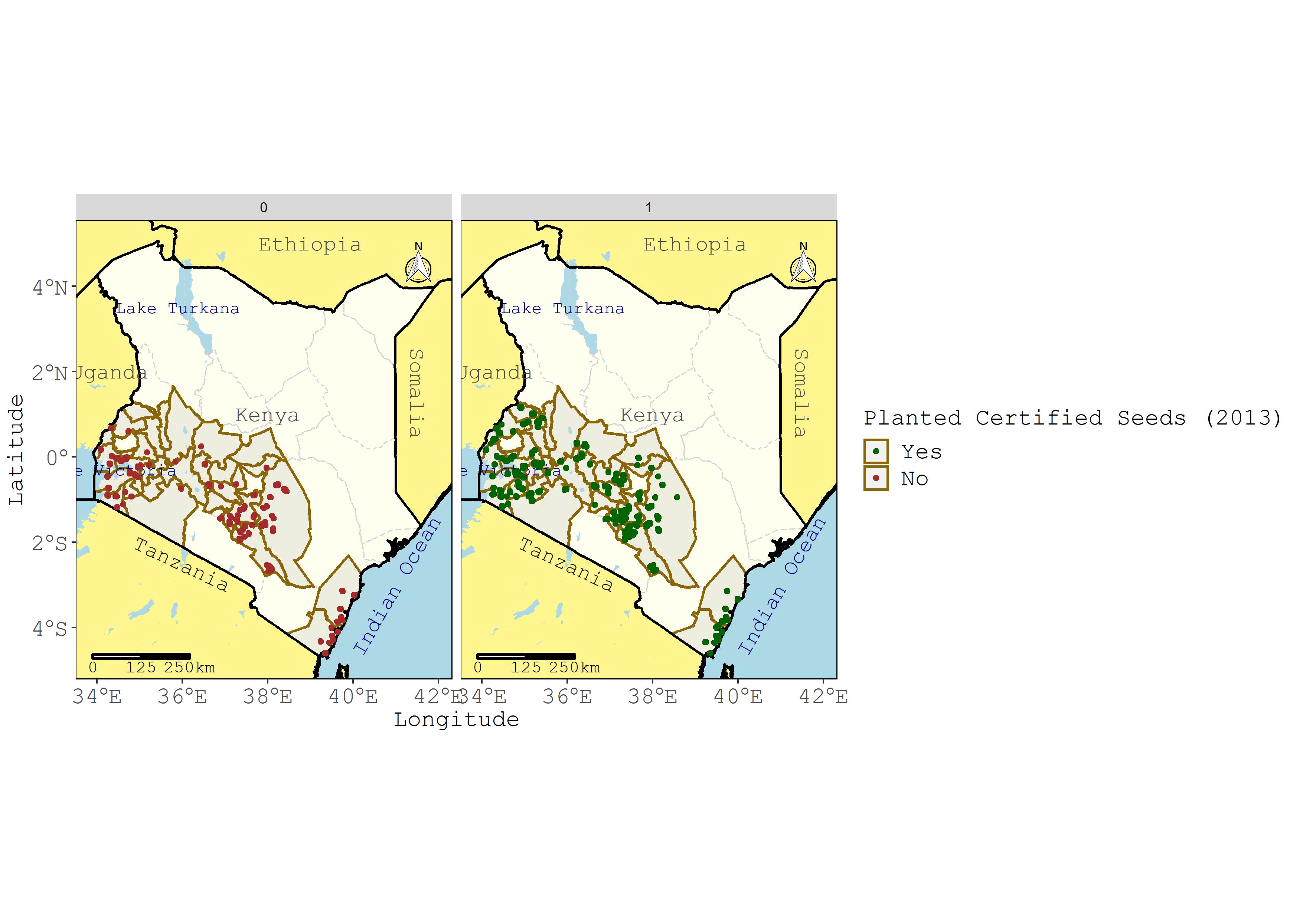


Supplementary Figure 16. Location of farmers who planted certified seeds or did not in 2013. The values 1 and 0 (title) are inputs to “Planted Certified Seeds” dummy variable for either case respectively. Generated using ggplot2 package (version 3.3.5) R version 4.1.2 (Rstudio version 2022.02.0+443 in windows 10).


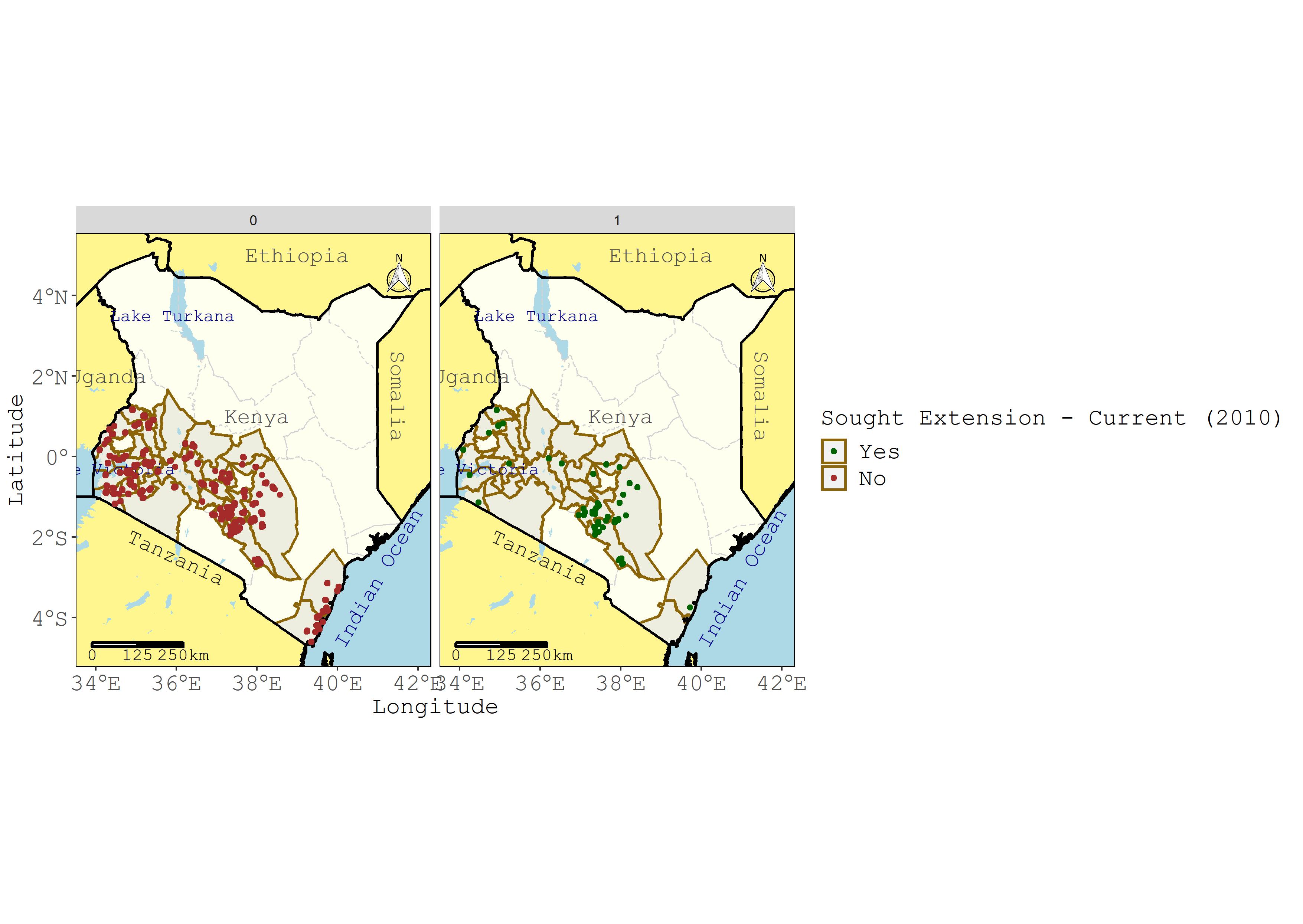


Supplementary Figure 17. Location of farmers who sought agricultural extension services or did not in 2010. The values 1 and 0 (title) are inputs to “Sought Extension - Current” dummy variable for either case respectively. Generated using ggplot2 package (version 3.3.5) R version 4.1.2 (Rstudio version 2022.02.0+443 in windows 10).


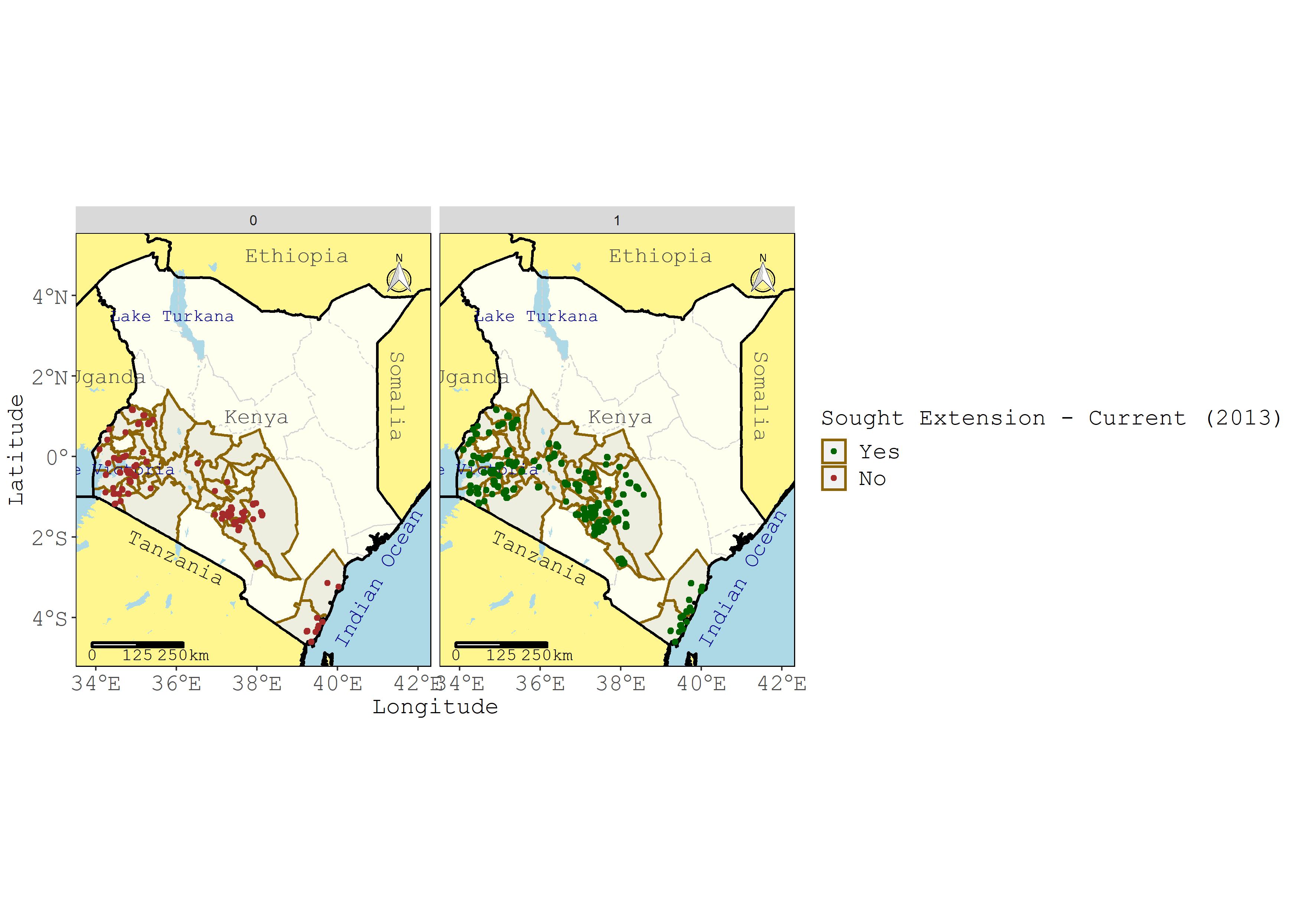


Supplementary Figure 18. Location of farmers who sought agricultural extension services or did not in 2013. The values 1 and 0 (title) are inputs to “Sought Extension - Current” dummy variable for either case respectively. Generated using ggplot2 package (version 3.3.5) R version 4.1.2 (Rstudio version 2022.02.0+443 in windows 10).


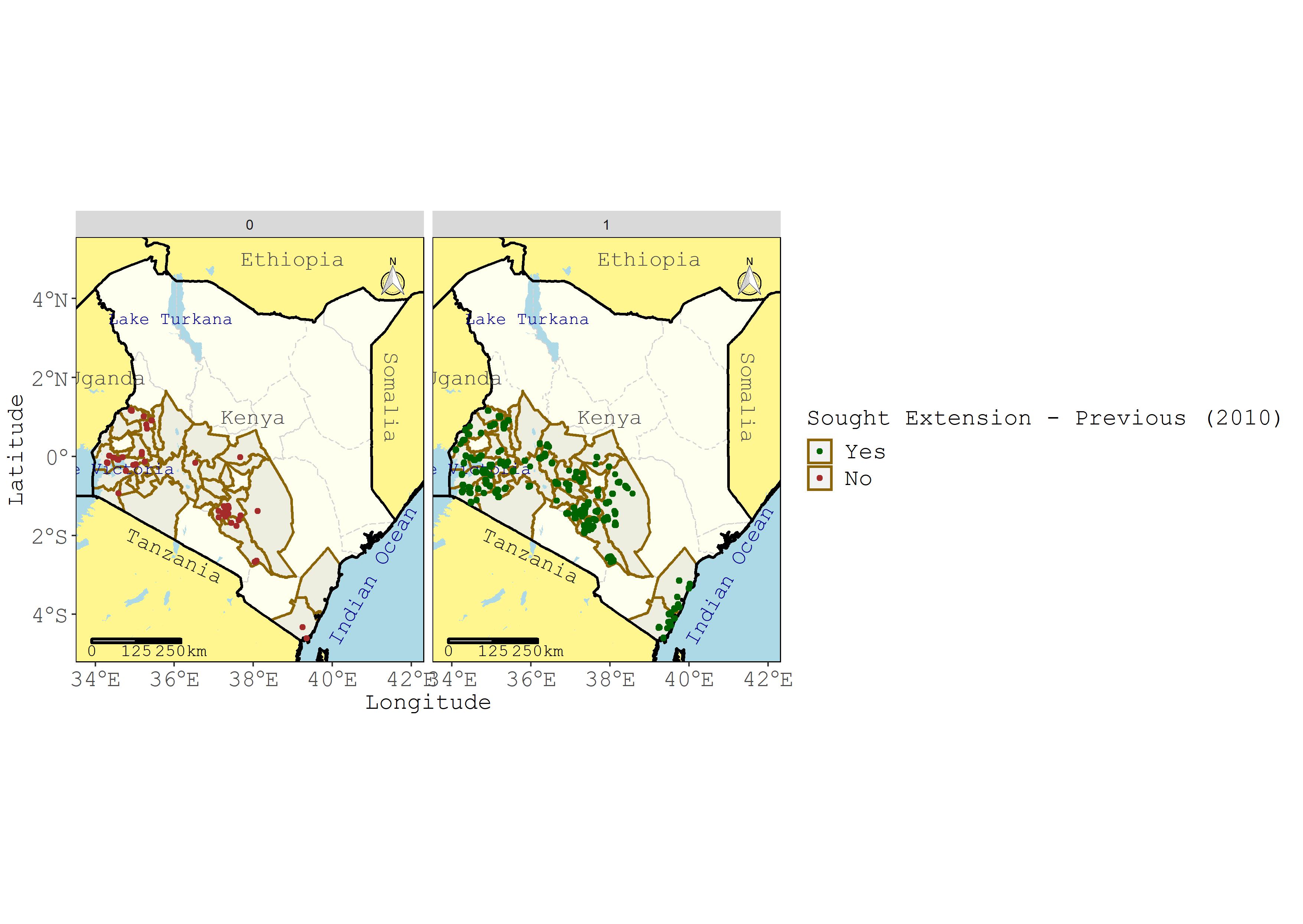


Supplementary Figure 19. Location of farmers who sought agricultural extension services or did not in a season prior to the 2010 season. The values 1 and 0 (title) are inputs to “Sought Extension - Pervious” dummy variable for either case respectively. Generated using ggplot2 package (version 3.3.5) R version 4.1.2 (Rstudio version 2022.02.0+443 in windows 10).


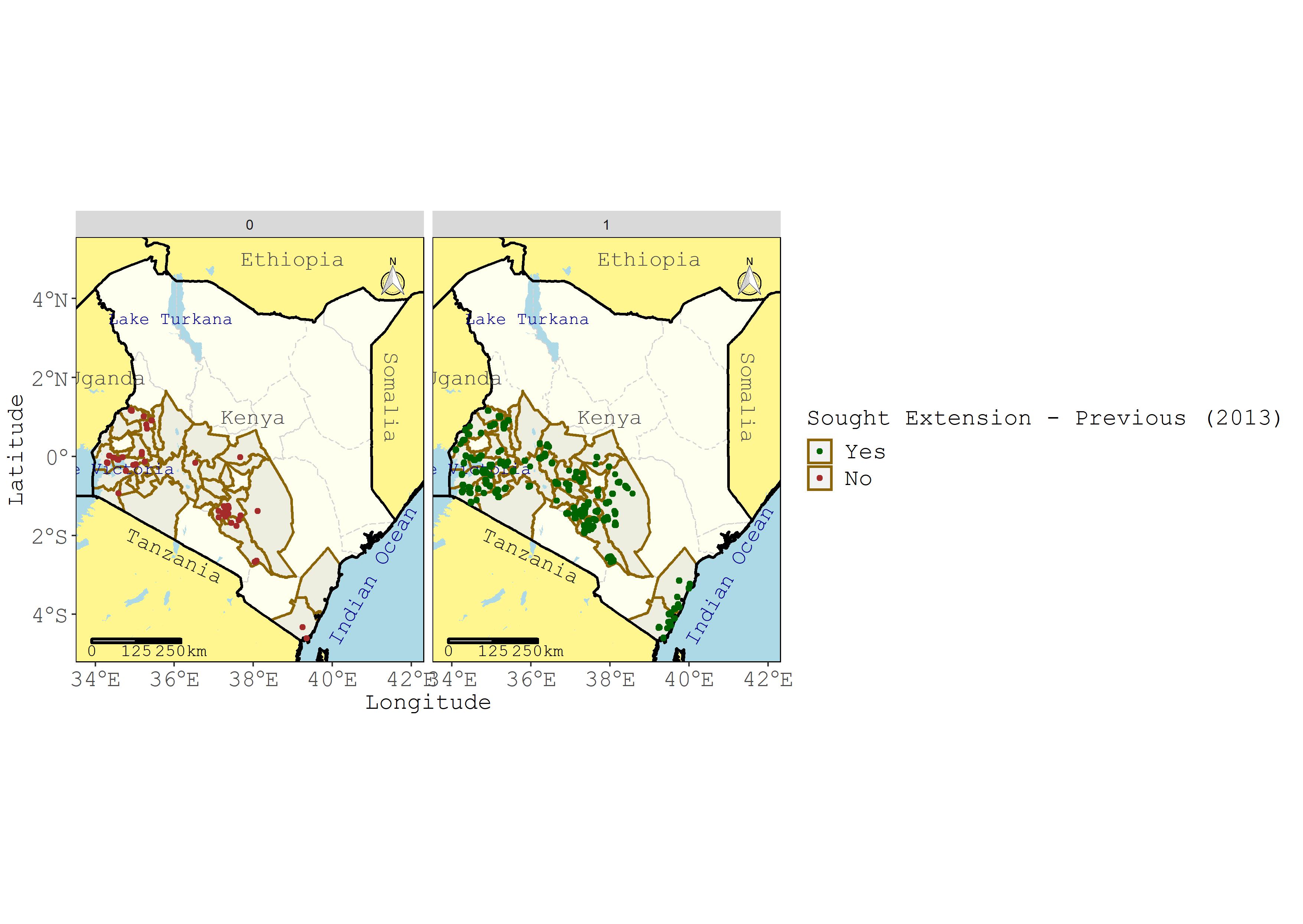


Supplementary Figure 20. Location of farmers who sought agricultural extension services or did not in a season prior to the 2013 season. The values 1 and 0 (title) are inputs to “Sought Extension - Pervious” dummy variable for either case respectively. Generated using ggplot2 package (version 3.3.5) R version 4.1.2 (Rstudio version 2022.02.0+443 in windows 10).


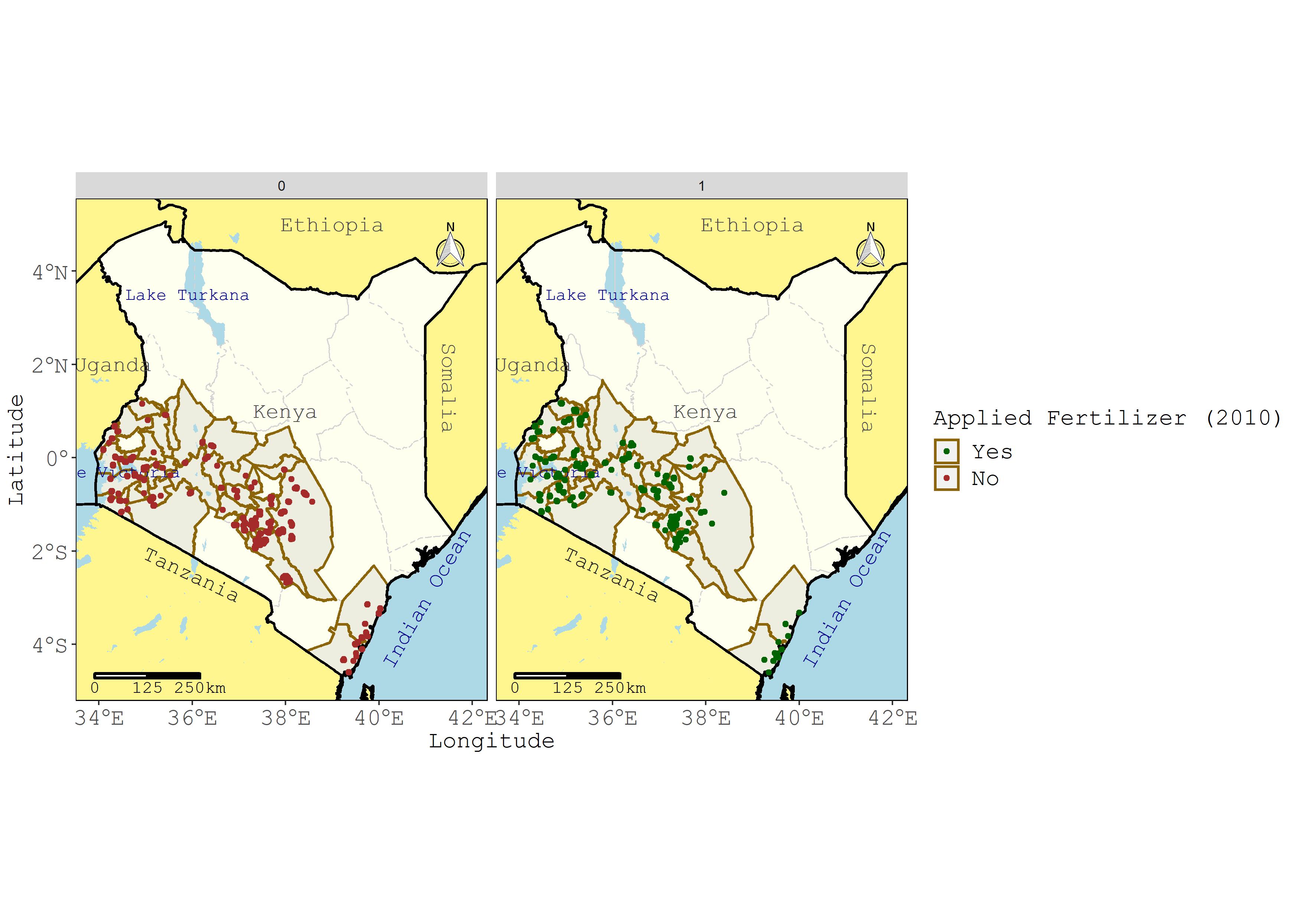


Supplementary Figure 21. Location of farmers who applied fertilizer or did not in 2010. The values 1 and 0 (title) are inputs to “Applied Fertilizer” dummy variable for either case respectively. Generated using ggplot2 package (version 3.3.5) R version 4.1.2 (Rstudio version 2022.02.0+443 in windows 10).


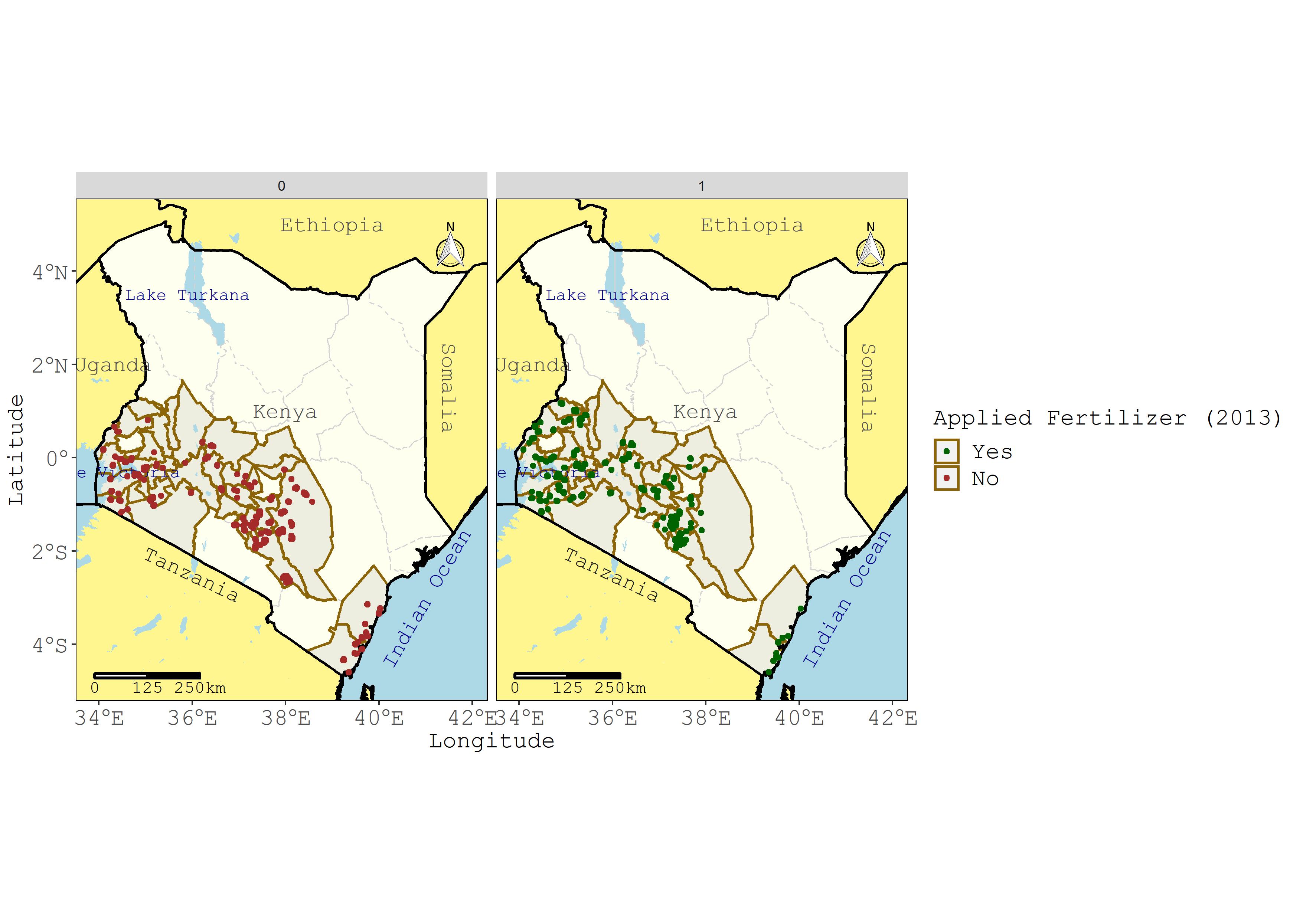


Supplementary Figure 22. Location of farmers who applied fertilizer or did not in 2013. The values 1 and 0 (title) are inputs to “Applied Fertilizer” dummy variable for either case respectively. Generated using ggplot2 package (version 3.3.5) R version 4.1.2 (Rstudio version 2022.02.0+443 in windows 10).


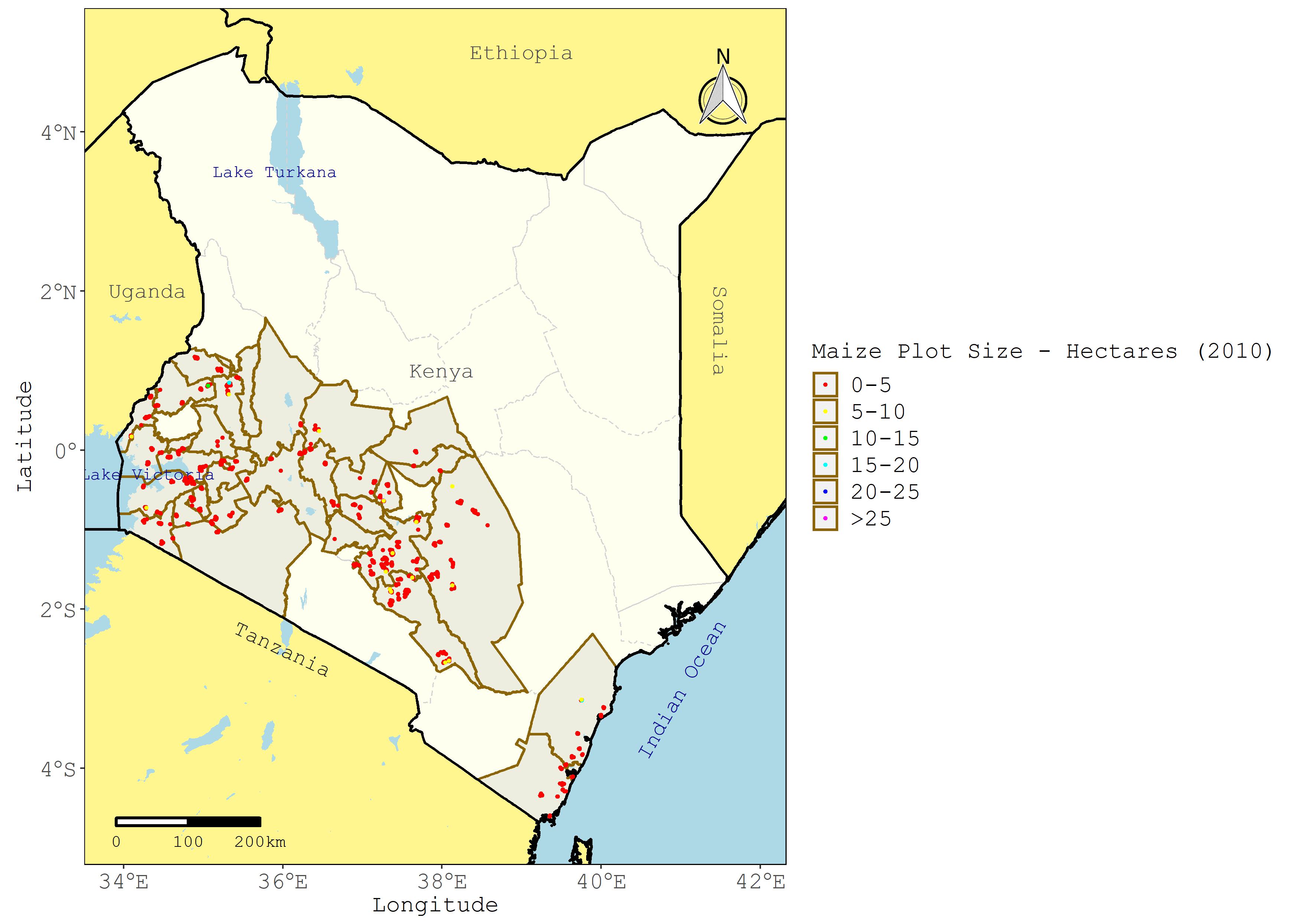


Supplementary Figure 23. Spatial distribution of area of plots under maize in 2010. Generated using ggplot2 package (version 3.3.5) R version 4.1.2 (Rstudio version 2022.02.0+443 in windows 10).


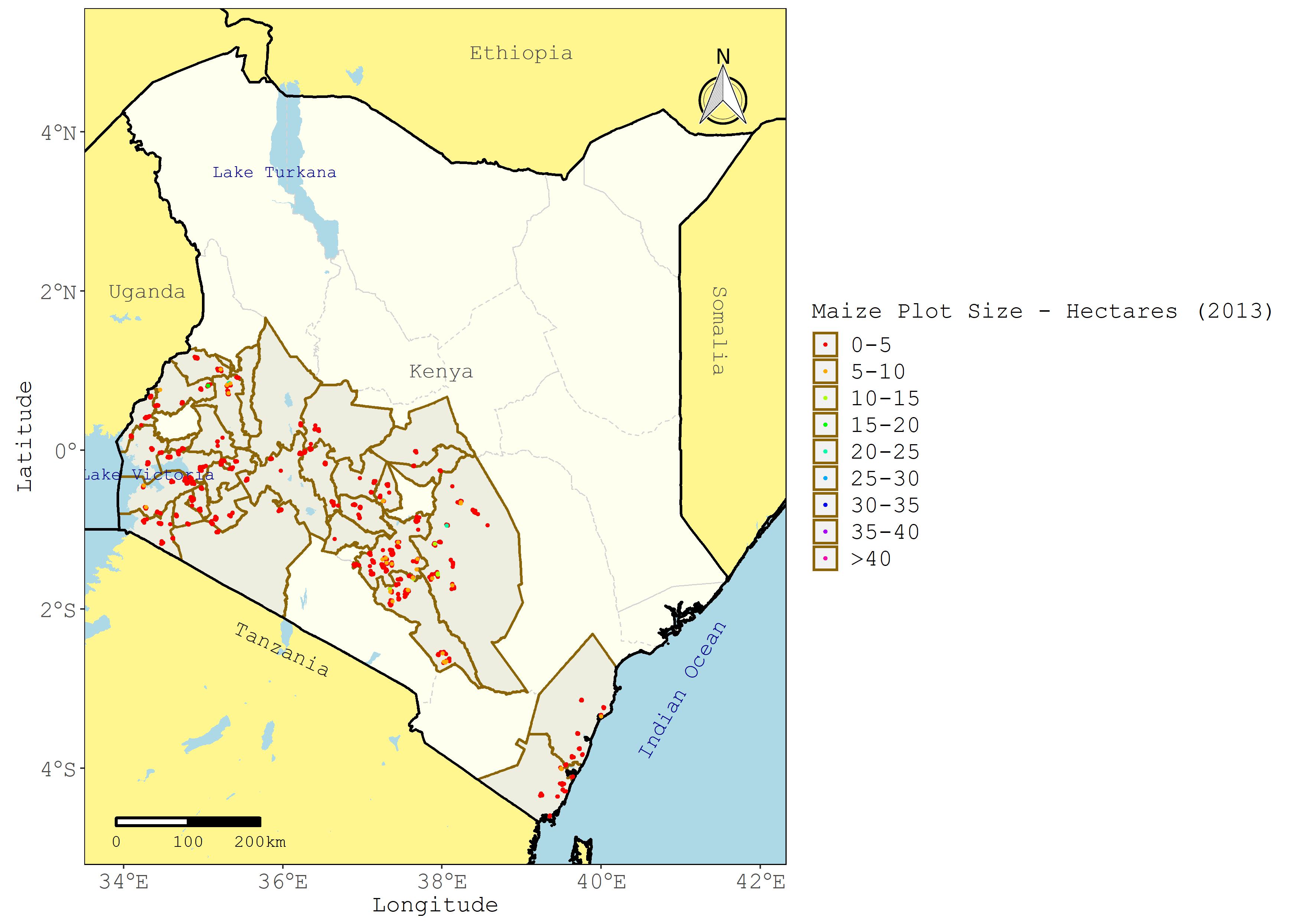


Supplementary Figure 24. Spatial distribution of area of plots under maize in 2013. Generated using ggplot2 package (version 3.3.5) R version 4.1.2 (Rstudio version 2022.02.0+443 in windows 10).


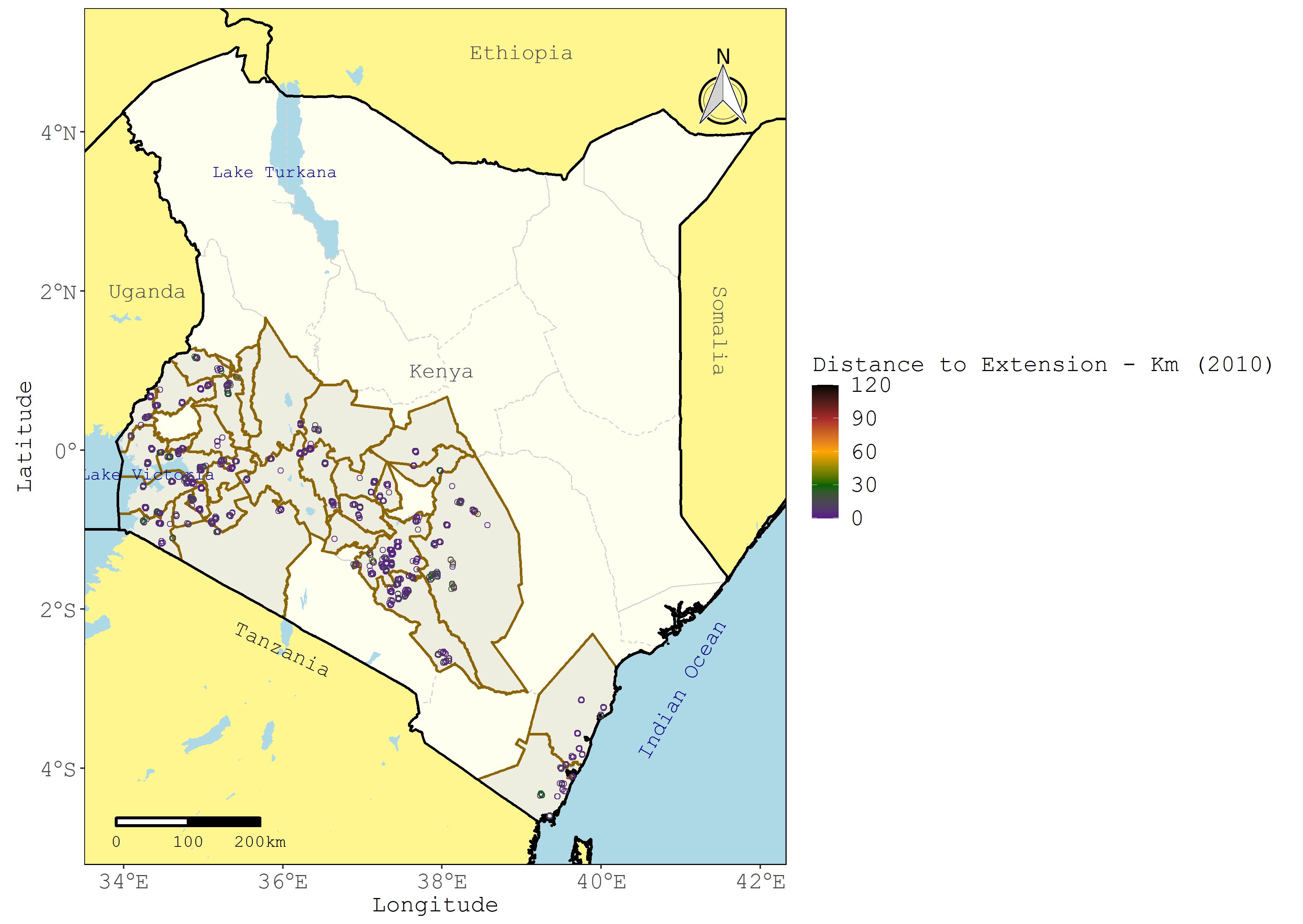


Supplementary Figure 25. Distribution of distances farmers must cover to access extension services in 2010. Generated using ggplot2 package (version 3.3.5) R version 4.1.2 (Rstudio version 2022.02.0+443 in windows 10).


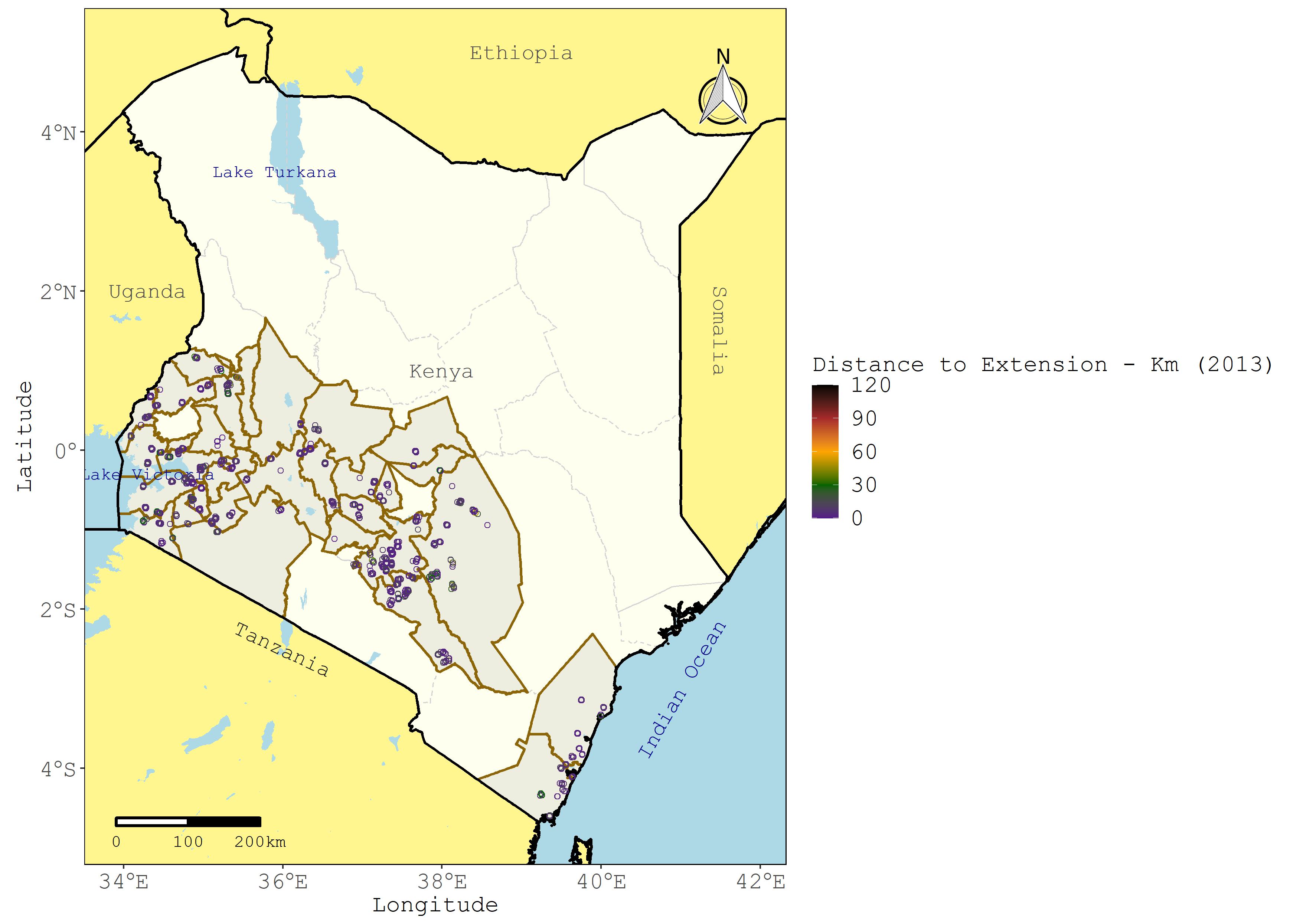


Supplementary Figure 26. Distribution of distances farmers must cover to access extension services in 2013. Generated using ggplot2 package (version 3.3.5) R version 4.1.2 (Rstudio version 2022.02.0+443 in windows 10).


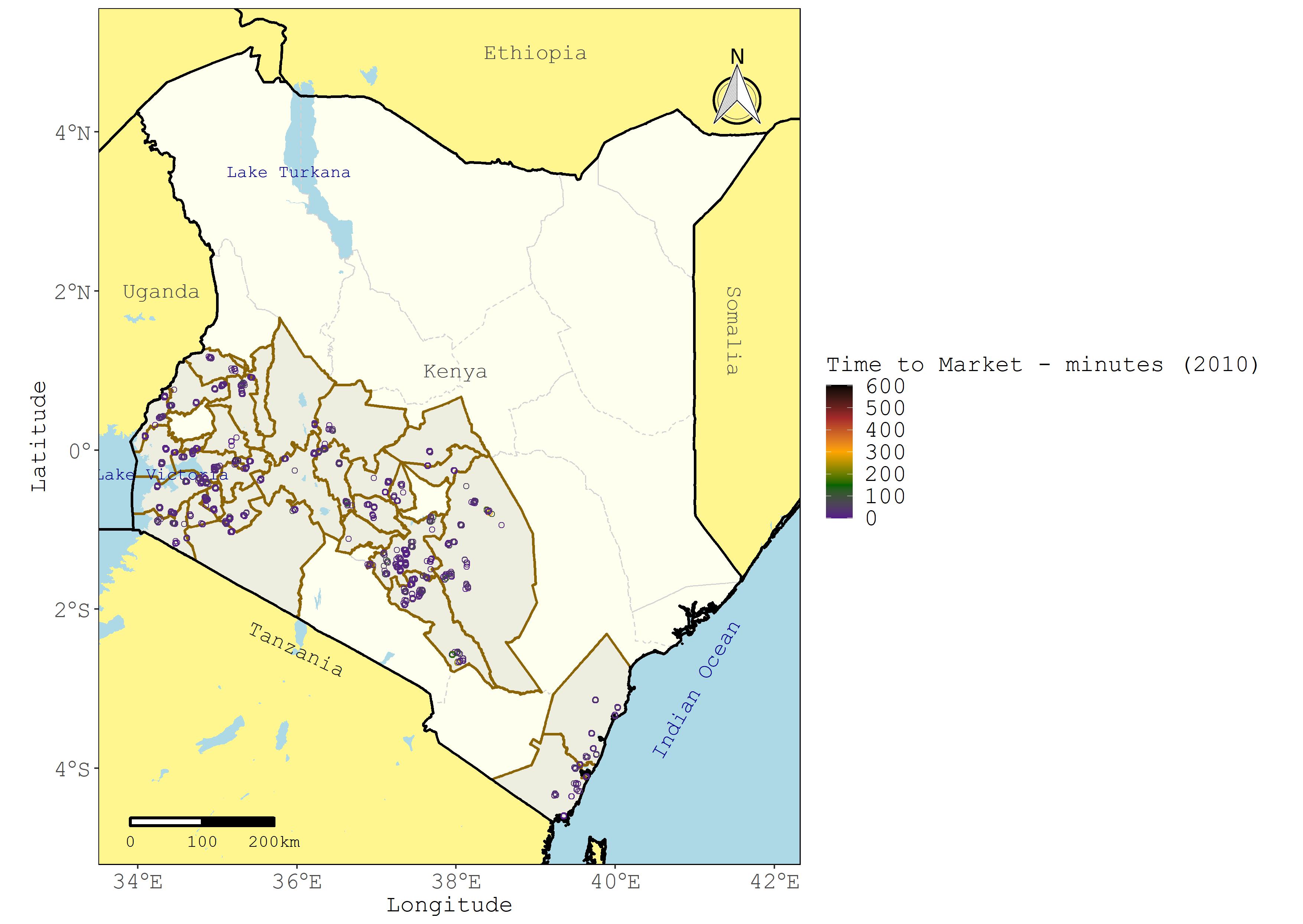


Supplementary Figure 27. Distribution of farmers’ time of travel to the market in 2010. Generated using ggplot2 package (version 3.3.5) R version 4.1.2 (Rstudio version 2022.02.0+443 in windows 10).


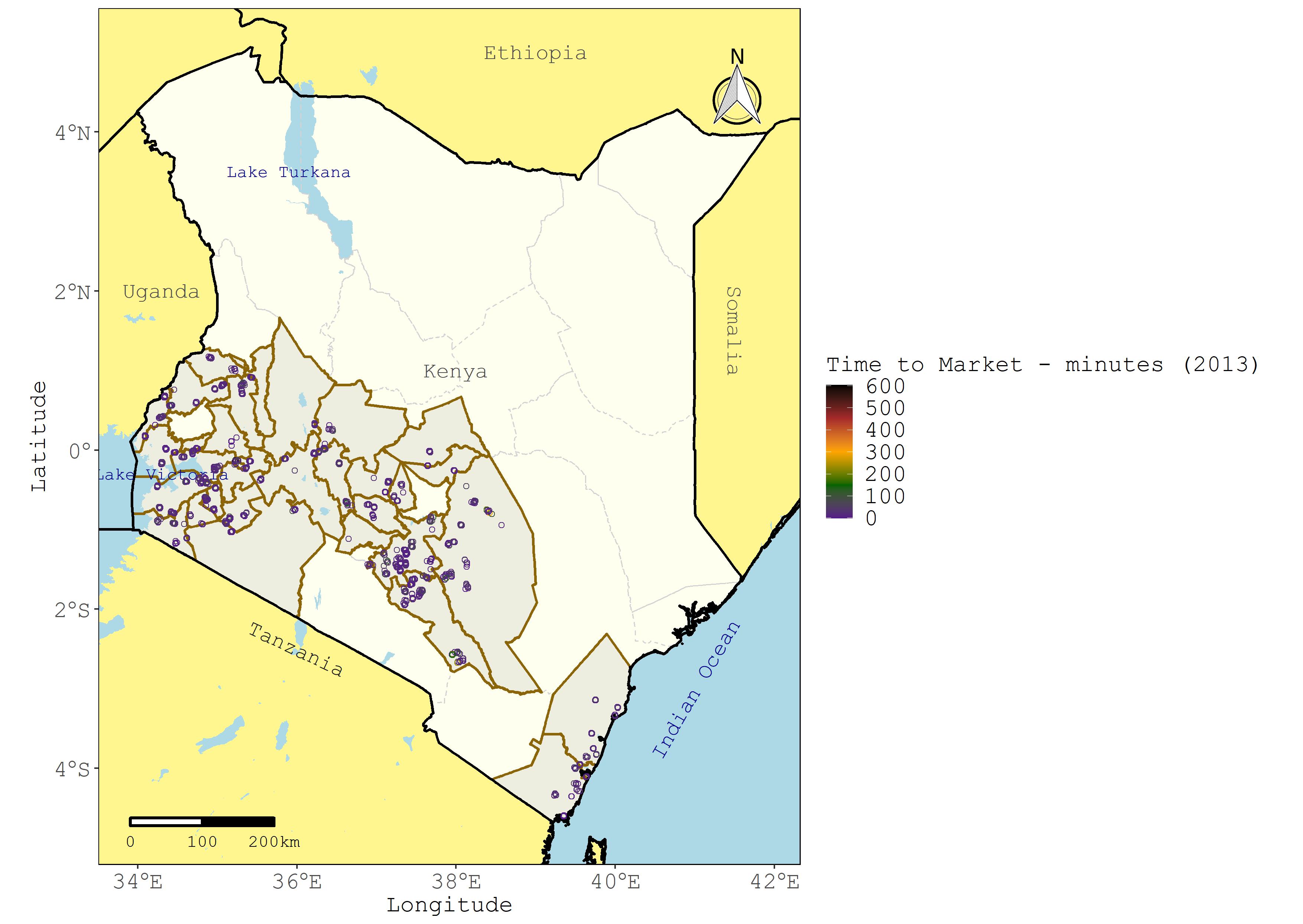


Supplementary Figure 28. Distribution of farmers’ time of travel to the market in 2013. Generated using ggplot2 package (version 3.3.5) R version 4.1.2 (Rstudio version 2022.02.0+443 in windows 10).


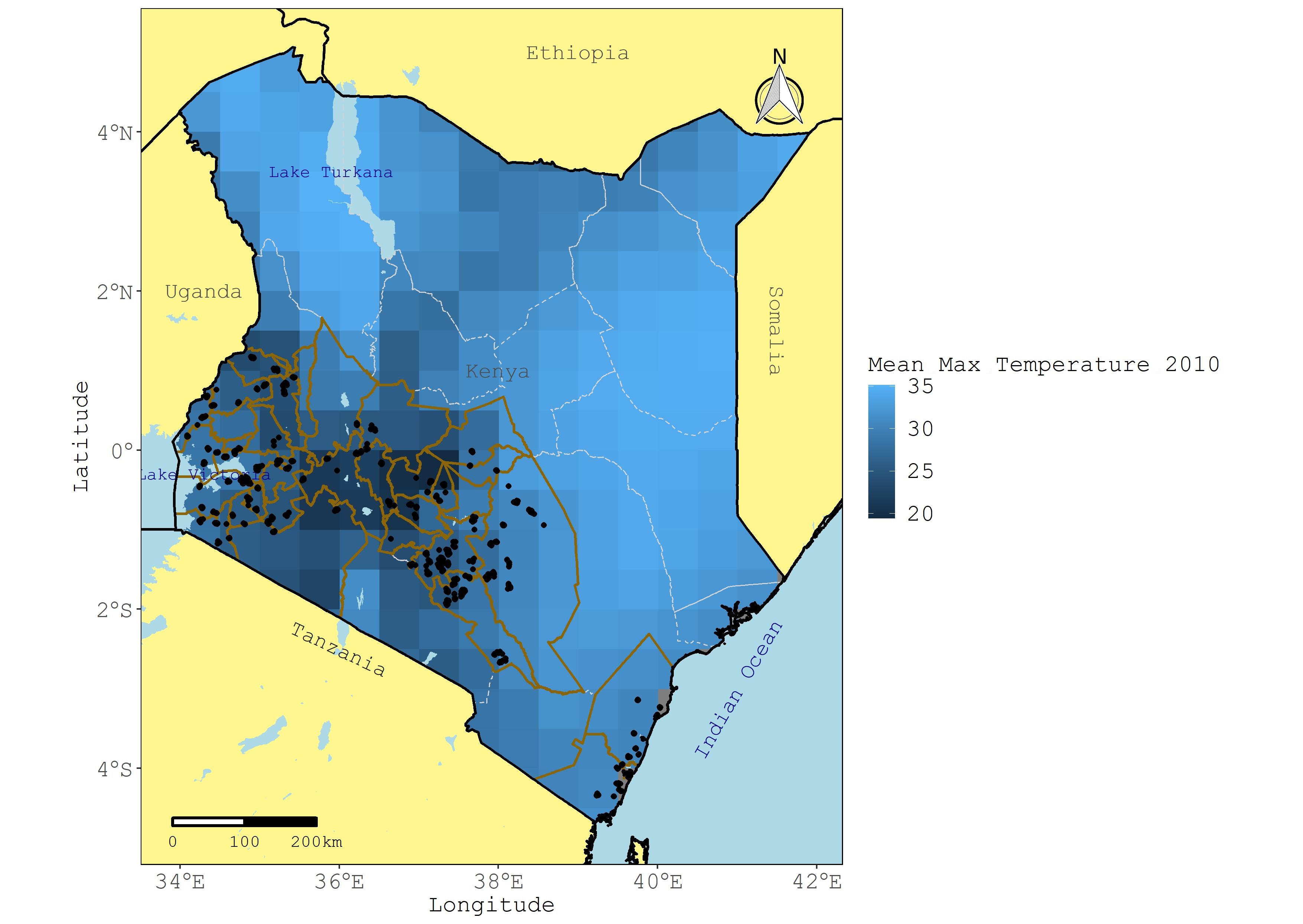


Supplementary Figure 29. Distribution of farmer locations on the mean daily maximum temperature (°C) surface in 2010. (CPC Global Temperature data provided by the NOAA/OAR/ESRL PSL, Boulder, Colorado, USA, from their Web site at https://psl.noaa.gov/data/index.html) Generated using ggplot2 package (version 3.3.5) R version 4.1.2 (Rstudio version 2022.02.0+443 in windows 10).


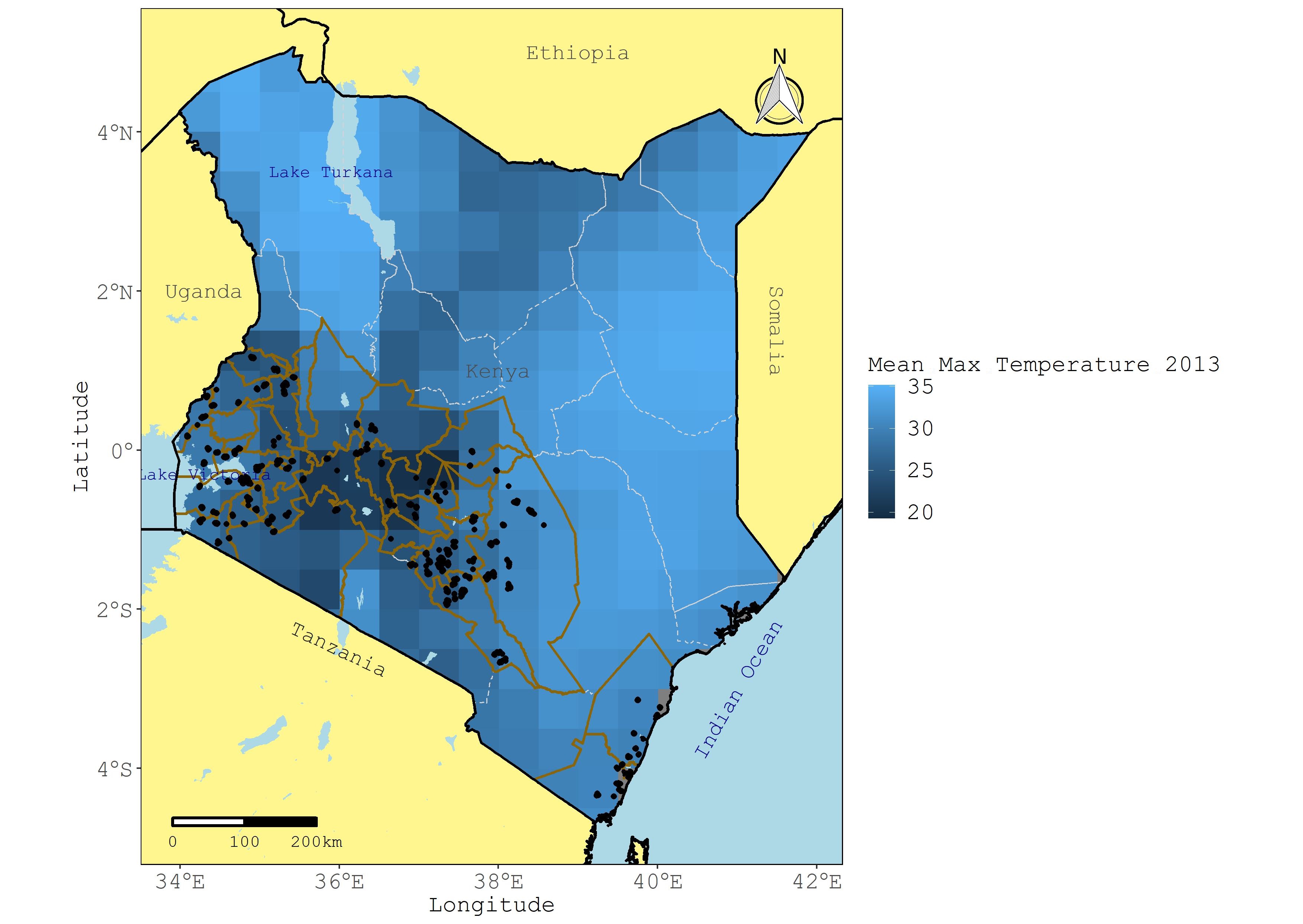


Supplementary Figure 30. Distribution of farmer locations on the mean daily maximum temperature (°C) surface in 2013 (CPC Global Temperature data provided by the NOAA/OAR/ESRL PSL, Boulder, Colorado, USA, from their Web site at https://psl.noaa.gov/data/index.html). Generated using ggplot2 package (version 3.3.5) R version 4.1.2 (Rstudio version 2022.02.0+443 in windows 10).


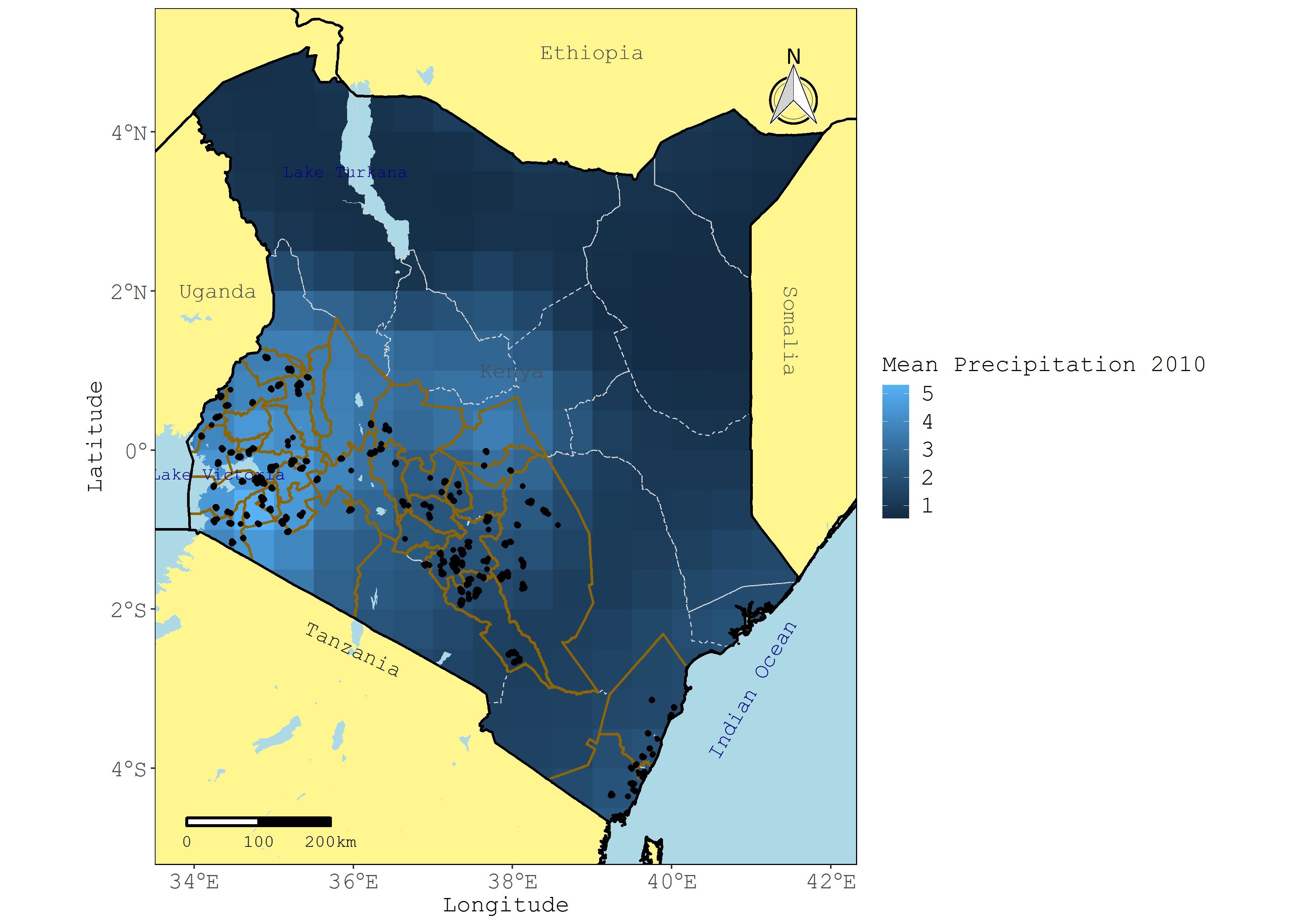


Supplementary Figure 31. Distribution of farmer locations on the mean daily precipitation (mm/day) surface in 2010 (CPC Global Precipitation data provided by the NOAA/OAR/ESRL PSL, Boulder, Colorado, USA, from their Web site at https://psl.noaa.gov/data/index.html). Generated using ggplot2 package (version 3.3.5) R version 4.1.2 (Rstudio version 2022.02.0+443 in windows 10).


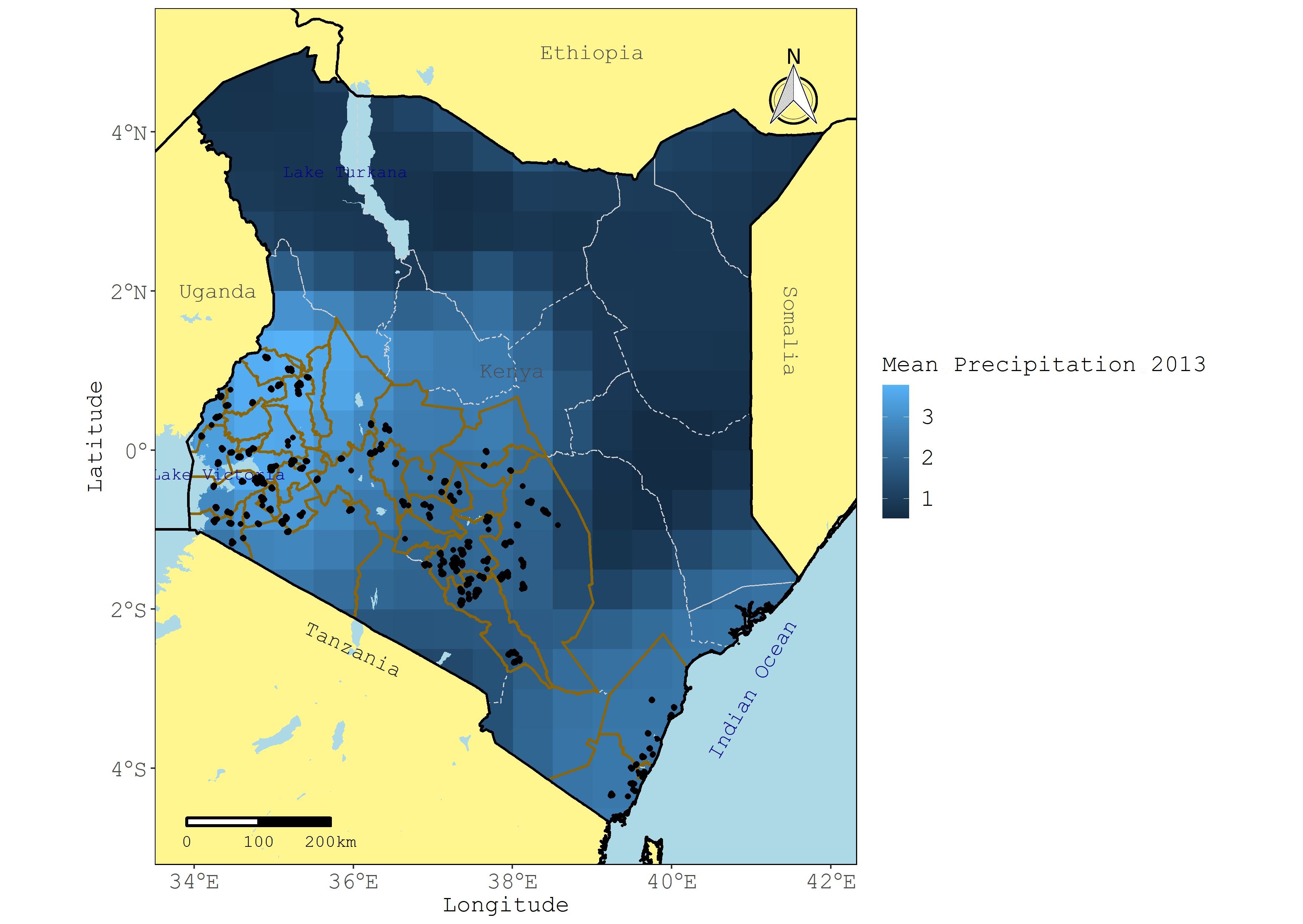


Supplementary Figure 32. Distribution of farmer locations on the mean daily precipitation (mm/day) surface in 2013 (CPC Global Precipitation data provided by the NOAA/OAR/ESRL PSL, Boulder, Colorado, USA, from their Web site at https://psl.noaa.gov/data/index.html). Generated using ggplot2 package (version 3.3.5) R version 4.1.2 (Rstudio version 2022.02.0+443 in windows 10).
